# Dietary Intervention Reverses Fatty Liver and Altered Gut Microbiota during Early-Life Undernutrition

**DOI:** 10.1128/mSystems.00499-20

**Published:** 2020-09-08

**Authors:** K. C. Bauer, K. E. Huus, E. M. Brown, T. Bozorgmehr, C. Petersen, M. S. Cirstea, S. E. Woodward, J. McCoy, J. Hun, R. Pamplona, V. Ayala, B. B. Finlay

**Affiliations:** a Michael Smith Laboratories, University of British Columbia, Vancouver, British Columbia, Canada; b Microbiology and Immunology Department, University of British Columbia, Vancouver, British Columbia, Canada; c The Metabolomics Innovation Centre, University of Victoria, British Columbia, Canada; d Institut de Recerca Biomèdica de Lleida (IRB-Lleida), Lleida, Spain; e Department of Metabolomic Physiology, Universitat de Lleida, Lleida, Spain; f Biochemistry and Molecular Biology Department, University of British Columbia, Vancouver, Canada; Vall d’Hebron Research Institute

**Keywords:** gut-liver axis, metabolomics, microbiome, NAFLD, undernutrition

## Abstract

Nonalcoholic fatty liver disease (NAFLD), largely studied as a condition of overnutrition, also presents in undernourished populations. Like NAFLD, undernutrition disrupts systemic metabolism and has been linked to gut microbiota dysbiosis. Indeed, chronic exposures to fecal microbes contribute to undernutrition pathology in regions with poor sanitation. Despite a growing prevalence of fatty liver disease, the influence of undernutrition and the gut microbiota remain largely unexplored. Here, we utilize an established murine model (C57BL/6J mice placed on a malnourished diet that received iterative Escherichia coli/*Bacteroidales* gavage [MBG mice]) that combines a protein/fat-deficient diet and iterative exposure to specific, fecal microbes. Fecal-oral contamination exacerbates triglyceride accumulation in undernourished mice. MBG livers exhibit diffuse lipidosis accompanied by striking shifts in fatty acid, glycerophospholipid, and retinol metabolism. Multiomic analyses revealed metabolomic pathways linked to the undernourished gut microbiome and hepatic steatosis, including phenylacetate metabolism. Intriguingly, fatty liver features were observed only in the early-life, but not adult, MBG model despite similar liver metabolomic profiles. Importantly, we demonstrate that dietary intervention largely mitigates aberrant metabolomic and microbiome features in MBG mice. These findings indicate a crucial window in early-life development that, when disrupted by nutritional deficiency, may significantly influence liver function. Our work provides a multifaceted study of how diet and gut microbes inform fatty liver progression and reversal during undernutrition.

**IMPORTANCE** Nonalcoholic fatty liver disease (NAFLD) remains a global epidemic, but it is often studied in the context of obesity and aging. Nutritional deficits, however, also trigger hepatic steatosis, influencing health trajectories in undernourished pediatric populations. Here, we report that exposure to specific gut microbes impacts fatty liver pathology in mice fed a protein/fat-deficient diet. We utilize a multiomics approach to (i) characterize NAFLD in the context of early undernutrition and (ii) examine the impact of diet and gut microbes in the pathology and reversal of hepatic steatosis. We provide compelling evidence that an early-life, critical development window facilitates undernutrition-induced fatty liver pathology. Moreover, we demonstrate that sustained dietary intervention largely reverses fatty liver features and microbiome shifts observed during early-life malnutrition.

## Abstract (precis)

Nonalcoholic fatty liver disease (NAFLD) remains a global epidemic, but it is often studied in the context of obesity and aging. Nutritional deficits, however, also trigger hepatic steatosis, influencing health trajectories in undernourished pediatric populations. Here, we report that exposure to specific gut microbes impacts fatty liver pathology in mice fed a protein/fat-deficient diet. We utilize a multiomics approach to (i) characterize NAFLD in the context of early undernutrition and (ii) examine the impact of diet and gut microbes in the pathology and reversal of hepatic steatosis. We provide compelling evidence that an early-life, critical development window facilitates undernutrition-induced fatty liver pathology. Moreover, we demonstrate that sustained dietary intervention largely reverses fatty liver features and microbiome shifts observed during early-life malnutrition.

## INTRODUCTION

Obesity-associated nonalcoholic fatty liver disease (NAFLD), with a global prevalence over 25%, remains a leading cause of liver disease around the world (1). While NAFLD incidence increases with age, recent studies estimate that NAFLD also affects 3 to 12% of the pediatric population (1, 2).

Up to 30% of NAFLD cases may progress to NASH (nonalcoholic steatohepatitis), a reversible condition characterized by hepatocellular ballooning, inflammation, and fibrosis (3, 4). As NASH advances, irreversible damage, including liver cirrhosis or hepatocellular carcinogenesis, may occur (5, 6). Linked to obesity and diabetes mellitus, NAFLD is considered both a driver and manifestation of metabolic syndrome, and it has been largely examined as a consequence of overnutrition (1, 7). Severe undernutrition, however, has also been shown to promote fatty liver through impaired lipid metabolism (8–10).

Over 10% of the worldwide population experiences undernutrition (11). Malnutrition accounts for nearly half of all deaths in children under the age of five (12). A critical, environmental burden contributing to the persistence of early-life malnutrition is fecal-oral contamination. Poor sanitation, including the lack of clean drinking water and access to hygienic toilet facilities, promotes chronic exposure to fecal microbes. These microbial exposures alter the gut microbiota and impair nutrient absorption (12–15). In pediatric populations, fecal-oral contamination and subsequent gut dysbiosis are linked to growth stunting and lasting malnutrition consequences, from neurocognitive impairment to poor metabolic function (14, 16–18).

Both undernutrition and gut microbes shape health trajectories, including systemic metabolic activity (12, 13, 16). Indeed, the gut microbiome has been implicated in fatty liver as changes in microbial composition and function, notably alterations of bacterium-mediated bile acid metabolism, have been reported in obese-associated NAFLD (5, 19). However, the precise influence of fecal-oral contamination on the etiology, pathology, and persistence of undernutrition-triggered fatty liver remains largely unexplored.

We previously reported chronic fecal exposures, via bacterial gavage, trigger growth faltering, gut dysbiosis, and broad metabolic alterations in malnourished mice—C57BL/6J mice placed on a malnourished diet that received iterative E. coli/*Bacteroidales* gavage (MBG) model (13, 20). The MBG bacterial gavage is comprised of specific fecal commensals (Escherichia coli/*Bacteroidales*). Subsequent MBG bacterial colonization not only reflects increased relative abundance of E. coli and *Bacteroidales* commensals reported in pediatric malnourished communities but also elicits gastrointestinal and immune features linked to fecal-oral contamination (13, 20–22). In an independent research study, colonization by E. coli and *Bacteroidetes* members, originally isolated from a malnourished pediatric microbiota, impaired weight gain in a murine model of malnutrition (21).

We have previously reported that E. coli/*Bacteroidales* exposures fail to robustly colonize the gut or trigger growth deficits in control (CON) mice. Furthermore, we demonstrated that enteric Salmonella enterica serotype Typhimurium infection increased hepatic lipidosis and inflammatory markers within the MBG liver, accompanied by a striking increase of hepatic *S.* Typhimurium burden. Enteric infection, however, failed to trigger immune and hepatic alterations in CON mice repeatedly exposed to E. coli/*Bacteroidales* (13). These reported findings indicate that the deleterious effects of E. coli/*Bacteroidales* fecal exposures require malnutrition. Whether fecal-oral contamination promotes fatty liver features in the absence of enteric insult remained undetermined.

Here, we show that exposure to specific fecal commensals (Escherichia coli/*Bacteroidales*) exacerbates triglyceride accumulation in the context of undernutrition. MBG livers display diffuse macrovesicular lipidosis accompanied by a striking shift in the liver metabolome, notably polyunsaturated fatty acid (PUFA) and retinol metabolism. Multiomic analyses linked phenylacetate and glycerophospholipid metabolism to hepatic steatosis and the MBG gut microbiome. Fatty liver histology was observed only in young mice, not in adult mice, exposed to MBG conditions. Importantly, we demonstrate that dietary intervention reversed fatty liver pathology and largely mitigated gut microbiota compositional and functional alterations, putatively driving undernutrition-induced fatty liver. Our work indicates a critical window of metabolic development that, when disrupted, may significantly impact liver function. Moreover, this study reveals the complexity of fatty liver pathology, characterizing a multihit model involving diverse metabolomic and microbial alterations. We anticipate that these findings will provide valued insights into the roles of diet and gut microbes in driving fatty liver pathology, highlighting potential therapeutic targets to address undernutrition-induced fatty liver.

## RESULTS

### Malnutrition and fecal-oral contamination promote fatty liver features.

**FIG 1:**
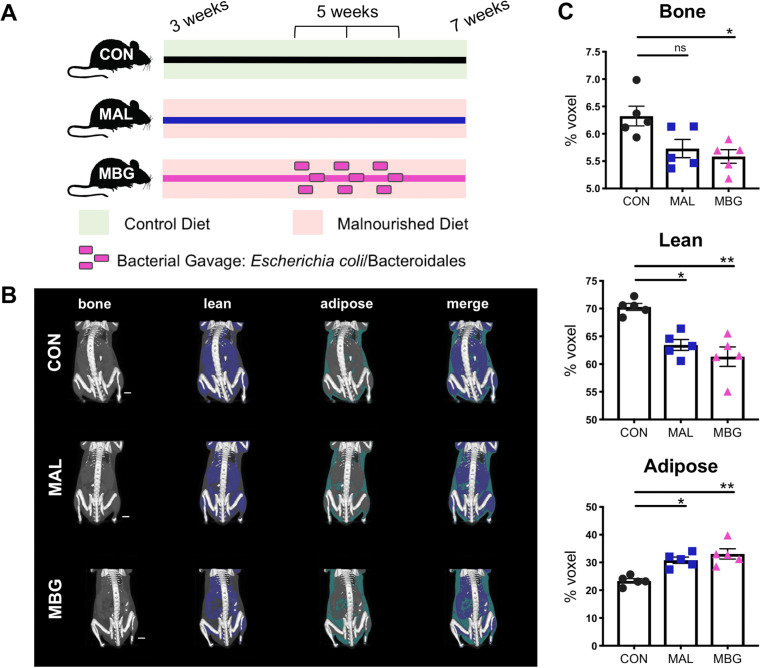
Malnourished model displays altered growth. (A) CON, MAL, and MBG experimental groups of 3-week-old mice were placed on control or malnourished diets. A subset of mice on the malnourished diet receive iterative bacterial gavage after 2 weeks (MBG mice). Liver features were examined 4 weeks following experimental start. The colors of the lines indicating diet and treatment are utilized to indicate murine groups in subsequent figures. (B) Representative images from reconstructed micro-CT scans. Mid-coronal plane with lean and adipose tissue highlighted in blue and green, respectively, is overlaid on surface-rendered bone tissue (white) (five mice per group). (C) Percent lean, bone, and adipose tissues from murine body (shoulder blade to tail). Bars indicate means ± standard errors of the means (SEM) (error bars). Statistical significance was determined by Kruskal-Wallis with *post hoc* Dunn’s test and indicated as follows: *, *P* < 0.05; **, *P* < 0.01; ns, not significant.

To explore the influence of diet and fecal gut commensals on liver function, we utilized the MBG murine model, an established malnutrition model previously characterized by our lab (13, 20). Briefly, newly-weaned C57BL/6J mice were placed on a malnourished diet (MAL mice). This protein/fat-deficient, carbohydrate-rich diet reflects dietary shifts previously reported in undernourished communities (13, 23–25). To model chronic fecal exposure, a subset of MAL mice received iterative E. coli/*Bacteroidales* gavage (MBG mice). Control (CON) mice, placed on a standard chow diet of equivalent caloric value, provided a healthy control (Fig. 1A).

We further characterized malnourished growth deficits with X-ray micro-computed tomography (micro-CT) (five mice per group). Following micro-CT scanning, three-dimensional images were reconstructed, and bone, lean, and adipose tissues were segmented using MicroView software (Fig. 1B). MAL and MBG mice exhibited a modest, albeit not significant, decrease in total volume (see Fig. S1A in the supplemental material). Fecal-oral contamination exacerbated growth alterations, notably loss of bone and lean body percentage. In contrast, both total volume and percentage of adipose tissues increased within MAL and MBG mice (Fig. 1B and C and Fig. S1), indicative of impaired nutrient storage and metabolism, a process requiring healthy liver function (26).

10.1128/mSystems.00499-20.2 FIG S1 MAL and MBG mice exhibit impaired growth. Total body volume (voxels) of CON, MAL, and MBG mice following micro-CT (*n *=* *5 per group). Download FIG S1, TIF file, 2.7 MB. Copyright © 2020 Bauer et al. 2020 Bauer et al. This content is distributed under the terms of the Creative Commons Attribution 4.0 International license.

**FIG 2:**
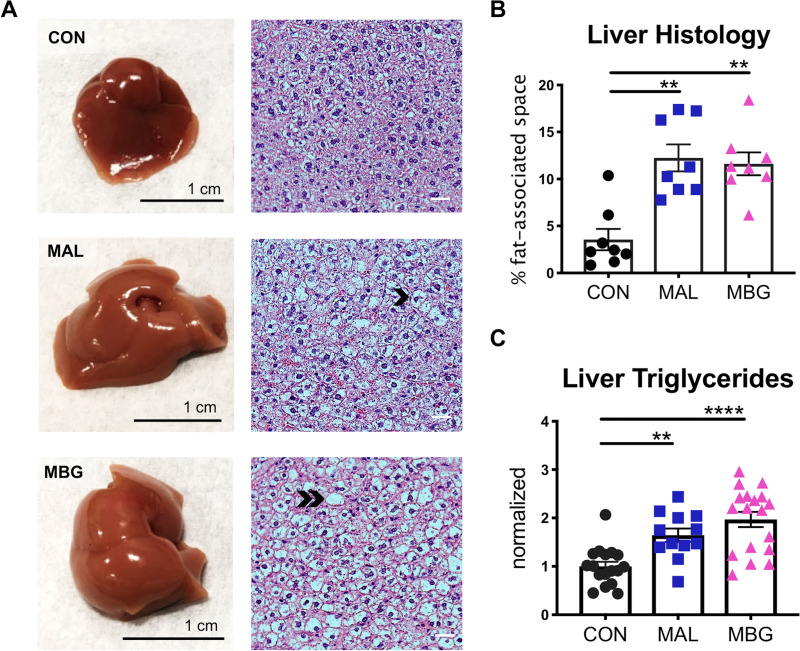
Malnutrition and fecal-oral contamination promote fatty liver features. (A) Representative whole liver (left) and H&E-stained liver histology (right). Malnourished livers exhibit microvesicular steatosis (hepatocyte lipid accumulation) and macrovesicular steatosis (hepatocyte lipid accumulation characterized by a large fat vacuole and displaced cell nucleus). Representative examples of microvesicular steatosis and macrovesicular steatosis are adjacent to the black chevron or double chevron, respectively. (B) Percent of fat-associated space (open spaces) in liver histology images, assessed with ImageJ software. Each point represents a biological sample. (C) Triglyceride level normalized to liver weight. Data were pooled from three experiments with triglyceride levels normalized to the CON group of each experiment. Bars indicate means ± SEM. Statistical significance was determined by Kruskal-Wallis with *post hoc* Dunn’s test (histology) or one-way ANOVA with *post hoc* Dunnett’s test (triglycerides) and indicated as follows: **, *P* < 0.01; ****, *P* < 0.0001.

After 4 weeks on the malnourished diet, MAL and MBG livers exhibit a paler appearance, suggestive of fatty liver (Fig. 2A). Despite visual discrepancies, liver weights and body-normalized liver weights were comparable across conditions (Fig. S2A). Hematoxylin and eosin (H&E) staining revealed diffuse hepatic steatosis throughout malnourished livers (Fig. 2A). While MAL and MBG mice exhibit comparable fat-associated space within the liver histology (fasted mice), fecal-oral contamination exacerbated hepatic fat/glycogen steatosis (nonfasted mice) and triglyceride levels during malnutrition (Fig. 2B and C and Fig. S2B). Despite fatty liver features, both MAL and MBG mice lacked histological evidence of significant steatohepatitis associated with NASH and inflammatory profiling revealed similar cytokine levels (gamma interferon [IFN-γ], interleukin 6 [IL-6], IL-12, monocyte chemotactic peptide 1 [MCP-1], and tumor necrosis factor alpha [TNF-α]) across CON, MAL, and MBG livers (Fig. S2C).

10.1128/mSystems.00499-20.3 FIG S2 Fatty liver and inflammatory profiling. (A) Liver weights and body-normalized liver weights and (B) percentage of fat/glycogen-associated space from H&E histological images. Data from four pooled mouse experiments are shown. (C) Proinflammatory cytokine levels were comparable within CON, MAL, and MBG livers, and samples were normalized to tissue weight. (D) Nonfasting (left) and fasting (right) insulin and glucose levels from mouse sera. Subsets of mouse experiments utilized in panel B were used for data presented in panels C and D. Bars indicate means ± SEM. Statistical significance as indicated in the legend to Fig. 4 was determined by Kruskal-Wallis with *post hoc* Dunn’s test (liver weight, histology, and inflammatory panel) or one-way ANOVA with *post hoc* Dunnett’s test (insulin, glucose). n.s., not significant. Download FIG S2, TIF file, 2.7 MB. Copyright © 2020 Bauer et al. 2020 Bauer et al. This content is distributed under the terms of the Creative Commons Attribution 4.0 International license.

As overnutrition-associated NAFLD and metabolic syndrome are highly connected (7), we also assessed clinical features of metabolic disruption. Insulin levels were comparable across groups under nonfasting and fasting conditions. While nonfasting mice exhibited comparable glucose concentrations, fasting glucose levels were elevated within MAL and MBG sera, possibly indicative of early insulin resistance and altered glucose metabolism (Fig. S2D). These results indicate that MAL/MBG mice display undernutrition-induced fatty liver features, with fecal-oral contamination promoting fat/glycogen-associated steatosis and impaired triglyceride metabolism.

**FIG 3:**
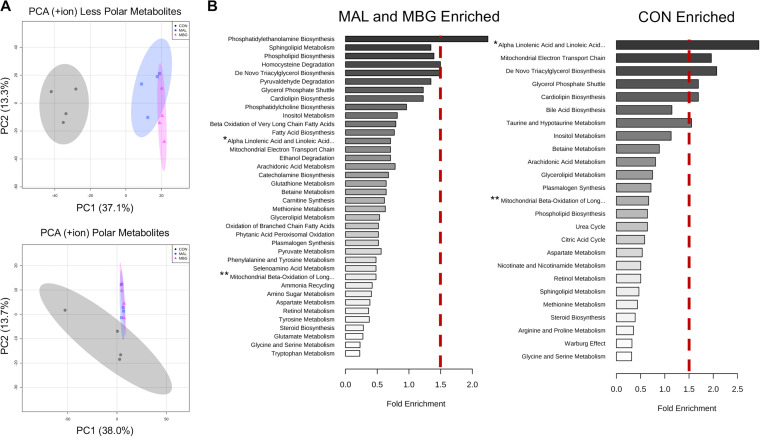
Malnutrition drives altered liver metabolome. (A) PCA plots of untargeted metabolomics via RP-UPLC-FTMS (top) and HILIC-FTMS (bottom). Data are from the positive ion channel (four mice per group). (B) Metabolite set enrichment analyses conducted with Metaboanalyst v 4.0 reported alterations in metabolomic pathways in the malnourished (MAL and MBG) and healthy (CON) liver. Metabolomic pathways to the right of the dashed red bar exhibit >1.5-fold enrichment compared to background metabolomic database. Fold enrichment is determined as the number of observed pathway hits divided by the number of expected hits. Untargeted metabolomics from the same experiment. The full metabolomic pathway names of the pathways with asterisks follow: *Alpha Linolenic Acid and Linoleic Acid Metabolism, **Mitochondrial Beta-Oxidation of Long-Chain Fatty Acids.

To further characterize metabolic shifts, we conducted untargeted metabolomics for less polar and polar metabolites via reversed-phase ultrahigh-performance liquid chromatography–Fourier transform mass spectrometry (RP-UPLC-FTMS) and hydrophilic interaction chromatography-FTMS (HILIC-FTMS), respectively, with four mice per group. Over 1,000 differentially abundant hits were detected following FTMS (one-way analysis of variance [ANOVA] Fisher’s least significant difference [LSD], adjusted *P* value [corrected for the false discovery rate] [*P*adj]* *<* *0.05). Of these hits, ∼350 differentially abundant metabolic features were annotated using the METLIN database. Diet predominantly shifted the liver metabolome as reported by unsupervised principal-component analysis (PCA) (Fig. 3A), and pathway analyses found no significantly enriched MAL versus MBG metabolomic pathways following false-discovery rate (FDR) correction (data not shown). Subsequent metabolite set enrichment analyses using Metaboanalyst 4.0 focused on dietary-driven metabolomic shifts (27).

Metabolite set enrichment analysis identified phosphatidylethanolamine (PE) biosynthesis, sphingolipid metabolism, and phospholipid biosynthesis as the top enriched metabolomic pathways identified in malnourished (MAL and MBG) livers (Fig. 3B), metabolic shifts observed in both undernutrition and hepatic steatosis models (13, 28). Compared to malnourished counterparts, CON livers exhibit enriched pathways linked to bile and PUFA metabolism, specifically α-linolenic acid (αLA) and linoleic acid (LA) metabolism (Fig. 3B), broadly reflecting shifts previously reported within the healthy small intestine (13).

**FIG 4:**
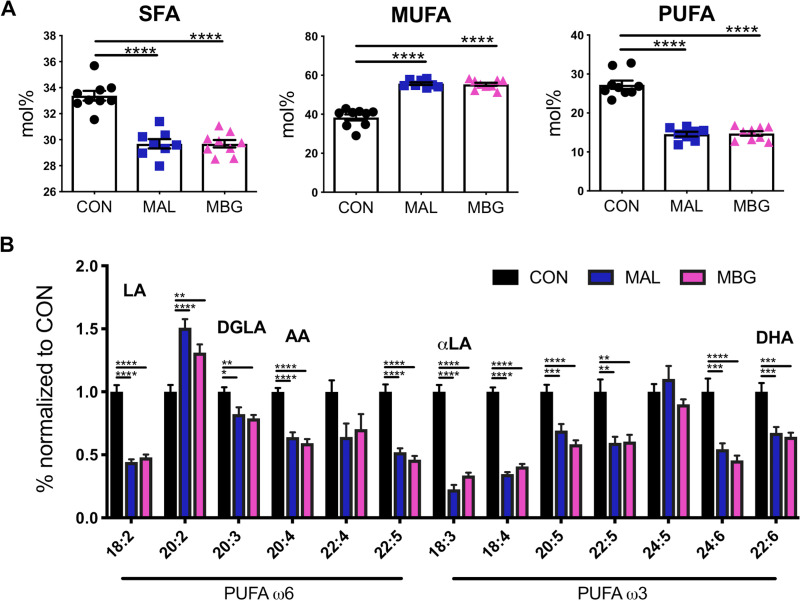
Malnutrition affects hepatic fatty acid liver profiles. (A) Fatty acid profiles in CON, MAL, and MBG livers determined by gas chromatography: total SFA, MUFA, and PUFA mol%. (B) The relative abundances of ω6 and ω3 PUFAs normalized to CON values are shown. In addition to LA and αLA, major PUFAs include DGLA (dihomo-γ-linolenic acid), AA (arachidonic acid), and DHA (docosahexaenoic acid). Fatty acid profiling from the same experiment. Bars indicate means ± SEM. Statistical significance was determined by ANOVA with *post hoc* Dunnett’s test and indicated by asterisks as follows: *, *P* < 0.05; **, *P* < 0.01; ***, *P* < 0.001; ****, *P* < 0.0001.

To extend untargeted metabolomic data, we conducted fatty acid profiling of total fatty lipids from liver tissue via gas chromatography. Malnourished mice displayed a reduction in saturated fatty acid (SFA) percent content (Fig. 4A). In contrast, malnutrition elevated relative unsaturated fatty acid (UFA) percent content (Fig. S3A). This increase was largely driven by monounsaturated fatty acid (MUFA) content, as MAL and MBG livers display nearly half the PUFA mol% of CON livers (Fig. 4A and B).

10.1128/mSystems.00499-20.4 FIG S3 Diet drives altered fatty acid metabolism. (A) The mol% of UFA identified by gas chromatography. (B) Ratios of ω6/ω3 hepatic PUFAs were comparable across groups. Fatty acid analyses were conducted from mice within the same experiment. Fatty acid bars indicate means and SEM with statistical significance determined by one-way ANOVA with *post-hoc* Dunnett’s test; significance indicated by asterisks is as reported in the legend to Fig. 4. Download FIG S3, TIF file, 2.7 MB. Copyright © 2020 Bauer et al. 2020 Bauer et al. This content is distributed under the terms of the Creative Commons Attribution 4.0 International license.

Dietary LA and αLA serve as precursors to downstream ω6 and ω3 PUFAs, respectively. MAL and MBG mice exhibit a persistent loss of LA and αLA metabolism supporting metabolic enrichment analyses (Fig. 3B and Fig. 4A and B). Elevated ω6/ω3 ratios—an inflammatory marker often associated with Western diets, have been associated with NALFD and NASH (29, 30). In our model, healthy and malnourished mice exhibit comparable ω6/ω3 ratios (Fig. S3B), further suggesting that moderate malnutrition may trigger hepatic steatosis uncoupled from significant inflammation.

### Dietary intervention largely reverses fecal microbiota shifts and fatty liver features.

We next sought to assess whether malnutrition and fecal-oral contamination intensifies fatty liver features specifically within early critical windows (childhood) or whether these perturbations trigger comparable fatty liver features in adult mice. Furthermore, we explored whether dietary intervention halts or reverses fatty liver pathology of early-life malnutrition.

**FIG 5:**
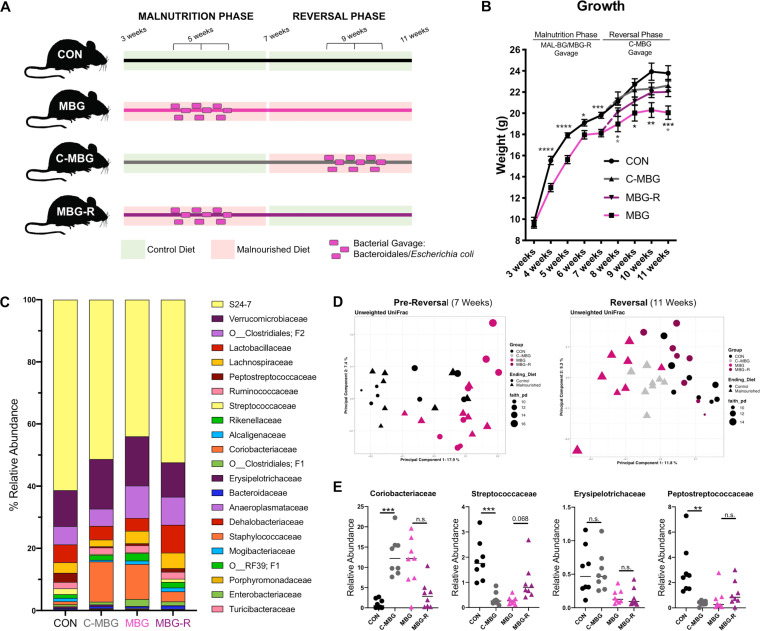
Dietary intervention improves growth deficits and alters gut microbiota composition. (A) Reversal experimental set-up: following 4 weeks on a healthy or malnourished murine chow diet, a subset of healthy and malnourished mice were “reversed” on the opposite diet to assess impacts of adult-onset malnutrition (C-MBG) and dietary intervention (MBG-R), respectively (eight mice per group). (B) Mouse weights across time. At 11 weeks, MBG-R mice exhibit a significant weight improvement. (C) 16S rRNA microbiota analyses from murine individual stool samples. Relative abundance of bacteria by family classification at the final time point is shown. (D) Unweighted UniFrac PCA with α-diversity (Faith’s phylogenetic diversity) of the CON and MBG microbiota (left) and the CON, C-MBG, MBG, and MBG-R microbiota (right). Each symbol represents the value for an individual mouse, and symbol shape reveals reversal diet. (E) Select bacterial family members at week 11. The short horizontal lines indicate the medians. Samples were from the same murine experiment. Microbiome analyses were conducted using QIIME2 (v. 2018.2). Statistical significance was determined by one-way ANOVA with *post hoc* Tukey’s test (growth) or Kruskal-Wallis with *post hoc* Dunn’s test in panel E and indicated by asterisks as in the legend to Fig. 4.

To address these unknowns, we conducted diet reversal experiments with eight mice per group. As MAL and MBG mice exhibited similar liver metabolomic profiles, we chose to utilize the MBG model during subsequent intervention studies. CON and MBG mice served as healthy and undernutrition-induced fatty liver controls, respectively. Following the initial 4-week phase, a subset of control mice was switched to the MBG model—C-MBG (adult-onset malnutrition)—while a subset of MBG mice received the control diet—MBG-R (reversal arm)—in order to assess the impact of dietary intervention on MBG fatty liver, model set-up reported in Fig. 5A.

After early-life malnutrition (7-week time point), MBG mice displayed significant weight faltering (*t* = 3.73, *P* = 0.0008). During the subsequent reversal phase, C-MBG mice exhibited a modest, but not significant, weight loss. The final weights of MBG-R mice, however, were comparable with those of CON and C-MBG mice by week 11 (F_3,28_ = 6.786, *P* = 0.0014; Fig. 5B). We observed no significant difference in tail size at week 11, suggesting that tail length differences previously reported in young malnourished mice (13), are largely resolved by adulthood and not influenced by adult-onset malnutrition (Fig. S4A).

10.1128/mSystems.00499-20.5 FIG S4 Diet and fecal-oral contamination influence growth and gut microbiota. (A) Final tail lengths at week 11. (B) Relative abundance of bacterial families determined by 16S rRNA sequencing at arrival (upon weaning), at week 7 (CON, MBG mice), and at week 11 (CON, C-MBG, MBG, and MBG-R) (*n *=* *8 per group). Columns represent microbiota composition (individual stool samples). (C) Relative abundance for select bacterial gavage members following the reversal phase. Data are from 16S rRNA analyses. (D) Box plot graph reporting unweighted UniFrac distances to CON mice at week 11. Postreversal unweighted UniFrac distance provides a measure of fecal microbiota similarity. C-MBG and MBG-R distances are between CON and MBG mice (permutational multivariate analysis of variance [PERMANOVA], pseudo-F = 2.12131, *P* = 0.001 [indicated by an asterisk]). The results of pairwise analyses are reported in Table S1. (E) Relative abundance of fecal microbiota of CON, C-MBG, MBG, and MBG-R groups by genus classification at week 11. (F) Differentially abundant microbiome pathways connote microbiome functionality. Fecal microbiome pathway analyses were determined by PICRUSt and annotated with the MetaCyc, reporting *Padj *<* *0.0002. The full PICRUSt output is shown in Table S2 (*n *=* *8 per group). Fecal microbiota composition was determined by 16S sequencing and analyzed with QIIME (v. 2018.2). Statistical significance was determined by one-way ANOVA with *post hoc* Tukey’s test (tail length) or Kruskal-Wallis with *post hoc* Dunn’s test in panel C. Data from murine samples of the same reversal experiment are shown. Bars in panels A and C indicate mean and SEM. n.s., not significant. In panel C, significance indicated by asterisks is as reported in the legend to Fig. 4. The asterisks in panel D indicate the lowest *P* value possible (*P* = 0.001) for 999 permutations. Download FIG S4, TIF file, 2.7 MB. Copyright © 2020 Bauer et al. 2020 Bauer et al. This content is distributed under the terms of the Creative Commons Attribution 4.0 International license.

As we introduce E. coli/*Bacteroidales* commensals in the initial malnutrition phase (MBG and MBG-R) and reversal phase (C-MBG), we also assessed fecal microbiota composition by 16S rRNA sequencing across time with fecal pellets taken upon weaning (arrival), after the initial malnutrition phase (week 7), and following reversal (week 11) and report the relative abundance of bacterial members by family classification (Fig. S4B and Fig. 5C). The relative abundance of specific bacterial gavage members was increased in C-MBG and/or MBG and/or MBG-R mice, but not CON animals (Fig. S4C).

PCA of unweighted UniFrac distances revealed distinct clustering by dietary group (CON and MBG) at week 7 (Fig. 5D). This MBG cohort exhibited increased α-diversity (Faith’s phylogenetic diversity [PD], Kruskal-Wallis: *H* = 7.71, *P* = 0.05; see Table S1 in the supplemental material). Upon reversal parameters, the fecal microbiota composition of C-MBG and MBG-R composition significantly shifted toward MBG and CON, respectively, as observed in unweighted UniFrac PCA and UniFrac distance metrics (Fig. 5D, Fig. S4D, and Table S1). Moreover, dietary intervention reduced MBG-R α-diversity, a pattern observed in CON counterparts, while C-MBG mice exhibited increased α-diversity (Faith’s PD, Kruskal-Wallis: *H* = 9.04, *P* = 0.03) (Table S1).

10.1128/mSystems.00499-20.8 TABLE S1 Microbiome analysis data set. Download Table S1, CSV file, 0.00 MB. Copyright © 2020 Bauer et al. 2020 Bauer et al. This content is distributed under the terms of the Creative Commons Attribution 4.0 International license.

Diet significantly influenced the relative abundance of select bacterial members with family and genus annotations reported in Fig. 5E and Fig. S4E. Bacteria from *Coriobacteriaceae* (*H* = 19.034, *P*adj* *=* *0.002) and *Streptococcaceae* families (*H* = 22.537, *P*adj* *=* *0.001) exhibit divergent shifts in response to malnourished diet. The relative abundance of *Coriobacteriaceae* species increased upon malnutrition, while dietary intervention somewhat mitigated *Coriobacteriaceae* bloom in MBG-R mice. Increased relative abundance of *Coriobacteriaceae* has been reported in rodent models following chronic stress (31, 32), suggesting that this bacterial family may be a marker of systemic strain. In contrast, the relative abundance of *Streptococcaceae* bacteria was decreased in malnourished mice (C-MBG and MBG), while MBG-R mice displayed increased relative abundance approaching CON abundance. Like *Streptococcaceae*, *Erysipelotrichaceae* bacteria have been linked with higher fat intake (33, 34). However, the relative abundance of *Erysipelotrichaceae* remained elevated in C-MBG mice and reduced in MBG-R mice (*H* = 16.366, *P*adj* *=* *0.005), matching the original, early-life diet. This finding may indicate that early-life malnutrition sets a long-term trajectory for *Erysipelotrichaceae* abundance, which is resilient against sustained dietary shifts during murine adulthood. Relative abundance of *Peptostreptococcaceae* was also increased in CON mice (*H* = 15.366, *P*adj* *=* *0.005), but showed a striking reduction in the C-MBG, MBG, and MBG-R gut microbiota, suggesting that bacteria within this family are highly sensitive to malnourished diet and may not recover, even after prolonged dietary intervention.

As these alterations may reflect model-specific bacterial shifts, we also predicted microbiome metabolic signatures of health and malnutrition using predictive PICRUSt analyses (35). Like compositional alterations, metabolic pathways of the C-MBG and MBG-R microbiota largely shifted toward MBG and CON counterparts, respectively, highlighting a robust microbial response to diet. Top differentially abundant PICRUSt hits following FDR correction included amino acid biosynthesis and degradation pathways, broadly matching metabolomic patterns between CON and MAL intestinal content (13). In addition, malnourished mice (MBG and C-MBG) exhibited elevated pathways contributing to the tricarboxylic acid cycle (TCA) (or citric acid cycle), potentially reflecting increased carbohydrate load in the MAL diet (Fig. S4F and Table S2).

10.1128/mSystems.00499-20.9 TABLE S2 PICRUSt data set. Download Table S2, CSV file, 0.3 MB. Copyright © 2020 Bauer et al. 2020 Bauer et al. This content is distributed under the terms of the Creative Commons Attribution 4.0 International license.

Fecal-oral contamination and diet drive lasting features of early-life malnutrition, notably physical stunting and gut dysbiosis. Our findings suggest that sustained dietary intervention considerably mitigates these consequences in our early-life malnourished model.

**FIG 6:**
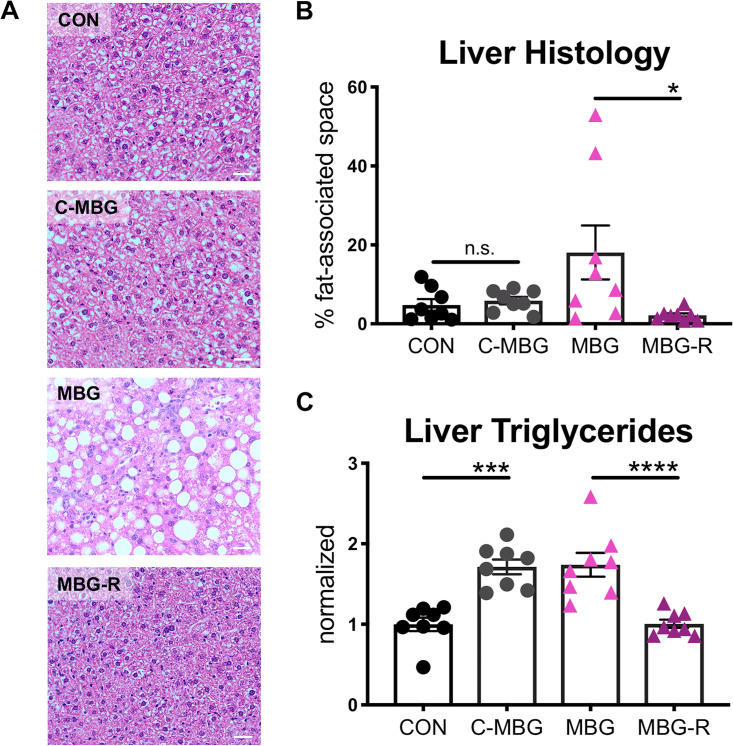
C-MBG and MBG-R mice lack fatty liver features. (A) Representative H&E staining of the liver following dietary reversal. (B) Percentage of fat/glycogen-associated space in nonfasted liver histology determined with ImageJ analysis. Each symbol represents the value for a biological sample. (C) Total triglyceride content in liver normalized to tissue weight. Data were normalized to CON levels. Analyses conducted from murine tissue of the same experiment. Bars indicate means ± SEM. Statistical significance was determined by one-way ANOVA with *post hoc* Tukey’s test and indicated as in the legend to Fig. 4.

Dietary reversal also mitigated fatty liver features. CON hepatocytes exhibited low-fat/glycogen content as observed by H&E staining, while 11-week-old MBG livers displayed increased hepatic macrovesicular lipidosis compared to 7-week-old MBG mice. Unexpectedly, undernutrition-induced fatty liver histology was not observed in either C-MBG or MBG-R livers (Fig. 6A and B). In contrast, triglyceride content was significantly elevated in both C-MBG and MBG mice, while triglyceride levels in MBG-R livers were comparable with those in CON mice (Fig. 6C).

**FIG 7:**
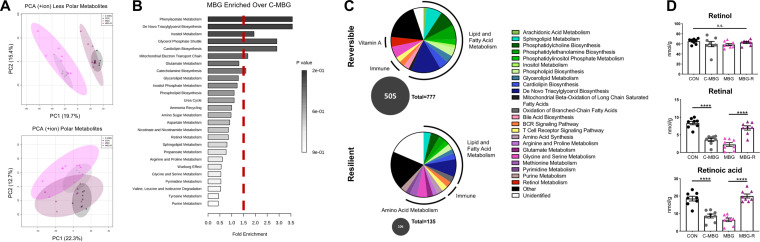
Dietary reversal significantly shapes liver metabolome. (A) PCA plots of untargeted liver metabolomics via RP-UPLC–FTMS (top) and HILIC-FTMS (bottom). Data from the positive ion channel are shown. (B) Metabolite set enrichment analyses conducted with Metaboanalyst v 4.0 (SMPDB database) reported enriched metabolomic pathways in the MBG versus C-MBG liver metabolome. Metabolomic pathways to the right of the dashed red bar exhibit >1.5-fold enrichment. Fold enrichment was determined as number of observed pathway hits divided by the number of expected hits. (C) Metabolomic pathway profiles of reversible (top) and resilient (bottom) metabolites. One-way ANOVA of metabolites with *post hoc* Fisher’s LSD revealed metabolites significantly altered between MBG-R and MBG but not MBG-R and CON (reversible) as well as metabolites altered between MBG-R and CON but not MBG-R and MBG (resilient). Metabolites were searched in SMPDB to identify biopathway(s). The numbers of metabolites belonging to reversible or resilient pathway is shown in a gray circle; the adjacent numeric value refers to the total number of pathways within each graph. Legend lists are from SMPDB pathways. (D) Retinol, retinal, and retinoic acid levels within hepatic livers conducted by targeted LC-MS and normalized to tissue weights. Analyses were conducted from mice of the same reversal study. Bars indicate means ± SEM. Statistical significance was determined by one-way ANOVA with *post hoc* Tukey’s test and indicated as in the legend to Fig. 4.

PCA of untargeted metabolomics for both less polar and polar metabolites confirmed a significant change in liver metabolomes, with the C-MBG and MBG-R metabolomic profile shifting toward MBG and CON, respectively (Fig. 7A). Over 2,000 differentially abundant hits were detected following RP-UPLC-FTMS and HILIC-FTMS analyses (one-way ANOVA Fisher’s LSD, *P*adj* *<* *0.05). Nearly 800 differentially abundant hits were putatively annotated by *m/z* values against the METLIN database.

We first explored metabolomic distinctions between chronic, early-onset malnutrition (MBG) and adult-onset malnutrition (C-MBG). While MBG and C-MBG livers exhibit largely similar metabolomic profiles (Fig. 7A), we identified metabolites elevated in persistent early malnutrition versus adult-onset malnutrition (45 metabolites: MBG enriched over C-MBG plus MBG enriched over CON) for metabolite set enrichment analyses. The top enriched pathway for the MBG profile was phenylacetate metabolism (Fig. 7B), a bacterial product from aromatic amino acid metabolism (AAAM) linked to hepatic steatosis (36). To explore whether the malnourished microbiome might contribute to enriched phenylacetic acid metabolism, we returned to predictive microbiome PICRUSt analyses. The MBG microbiome exhibits significantly elevated AAAM pathways compared to healthy controls (ARO-PWY, *P*adj* *=* *0.001; COMPLETE-ARO-PWY, *P*adj* *=* *0.002). While these predicted pathways did not reach statistical significance postreversal in CON, C-MBG, MBG, and MBG-R samples (ARO-PWY, *P*adj* *=* *0.105; COMPLETE-ARO-PWY, *P*adj* *=* *0.078), the relative frequency of AAAM pathways was higher in the MBG microbiome than in the C-MBG microbiome, as well as the microbiome of mice fed a healthy diet (CON and MBG-R), indicating a putative causal role for the MBG microbiota in undernourished-induced fatty liver (Fig. S5).

10.1128/mSystems.00499-20.6 FIG S5 Dietary reversal affects predictive AAAM pathways. Relative frequency (as a percentage) of AAAM microbiome pathways prior to and following reversal studies, with *P*adj reported. Functional microbiome analyses were conducted via PICRUSt (35). Download FIG S5, PPTX file, 0.2 MB. Copyright © 2020 Bauer et al. 2020 Bauer et al. This content is distributed under the terms of the Creative Commons Attribution 4.0 International license.

We then categorized MBG-R metabolomic features as “reversible” or “resilient” to dietary intervention. We considered reversible metabolites as those significantly different between MBG-R and MBG but not MBG-R and CON, while resilient metabolites were significantly altered between MBG-R and CON but not MBG-R and MBG. Of the differentially abundant metabolite hits, 505 were categorized as reversible, while only 106 metabolite features were considered resilient, supporting robust reversal of the fatty liver metabolome upon dietary intervention (Fig. 7C). Metabolites were then classified and grouped into metabolomic pathways using the small molecule pathway database (SMPDB). Adaptive immune pathways—B cell receptor (BCR) signaling pathway and T cell receptor signaling pathway—were observed in both reversible and resilient metabolomic profiles. The resilient profile featured many pathways associated with arachidonic acid (AA) metabolism within the liver. In contrast, the reversible profile included metabolites contributing to retinol (vitamin A_1_) metabolism. Moreover, nearly 60% of reversible metabolites were involved in lipid and fatty acid metabolism, notably amino acid metabolism and various phospholipid biosynthesis pathways (Fig. 7C).

To confirm untargeted metabolomic profiling, we assessed vitamin A metabolites and profiled long-chain fatty acids following dietary reversal. While the healthy and malnourished diets have distinct macronutrient profiles (e.g., reduced fat, elevated carbohydrates), both diets contain identical micronutrient content, including vitamin availability (13). Following absorption within the small intestine, dietary retinol can be esterified into retinyl ester (storage form) or oxidized to retinal and retinoic acid (37). We quantified retinoid levels within murine liver tissue with liquid chromatography (LC)-MS. While retinol levels were comparable across groups, malnourished mice (MBG and C-MBG) displayed reduced retinal and retinoic acid levels. As expected from metabolomic pathway analyses, dietary intervention mitigated retinoid shifts in MBG-R mice (Fig. 7D), supporting a reversible metabolic pattern.

**FIG 8:**
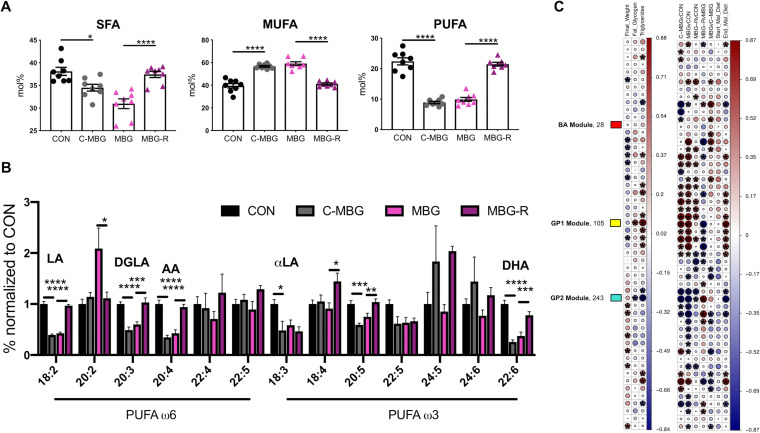
Altered long-chain fatty acid and glycerophospholipid metabolism associated with hepatic steatosis. (A) mol% of SFA, MUFA, and PUFA liver content assessed by gas chromatography. (B) Relative abundances of ω6 and ω3 PUFAs normalized to CON values. (C) WGCNA results. (Left) Associations between metabolomic clusters and clinical traits; (right) associations between group and diet (right). Start_Mal_Diet and End_Mal_Diet describe malnourished versus healthy diet, i.e., MBG/MBG-R versus CON/C-MBG and MBG/C-MBG versus CON/MBG-R, respectively. The size and color of the circle represent the Spearman correlation coefficient between metabolomic module and clinical/group traits with significance (*P*adj * *<* *0.05). WGCNA randomly assigned modules a color name. Three modules were annotated for further analyses: (i) “yellow/GP1” (positive correlation with hepatic steatosis: glycerophopholipid enriched), (ii) “turquoise/GP2” (negative correlation with hepatic steatosis: glycerophospholipid and SFA enriched), and (iii) “red/BA” (no correlation; bile acid enriched). Data in panels A, B, and C are from the same mouse reversal experiment (eight mice per group). Bars indicate means ± SEM with statistical significance determined by ANOVA with *post hoc* Tukey’s test. Further WGCNA findings are in Table S3 and Text S1 in the supplemental material.

As altered retinol metabolism also influences fatty acid metabolism (38), we assessed long-chain fatty acid profiles following dietary reversal. Fatty acid profiles of 11-week-old CON and MBG livers exhibit patterns similar to those of their 7-week counterparts (Fig. 4A and B and Fig. S3A). As expected, SFA and PUFA percent content remained elevated within healthy livers and reduced in malnourished mice, while the MBG liver displayed increased MUFA mol% (Fig. 8A). The reversal (end) diet, rather than early-life diet, shaped fatty acid content as C-MBG and MBG livers exhibited fatty acid profiles similar to those of MBG and CON livers, respectively (Fig. 8A and B and Fig. S6A). We specifically assessed whether dietary intervention increased the relative abundance of ω6 and ω3 PUFAs within the liver. Decreased mol% of LA and LA-derived ω6 PUFAs in malnourished mice (C-MBG and MBG mice) were reversed upon dietary intervention (MBG-R mice). While αLA relative abundance was elevated in CON livers, dietary intervention failed to shift αLA percent content in MBG-R mice. Dietary intervention, however, reversed shifts in downstream PUFA ω3 members, including 20:5 ω3 and 22:6 ω3 (docosahexaenoic acid [DHA]). Despite reported PUFA alterations, ω6/ω3 PUFA ratios remained comparable across all groups following dietary reversal (Fig. 8B and Fig. S6B).

10.1128/mSystems.00499-20.7 FIG S6 Long-chain fatty acid and glycerophospholipid metabolism linked to undernourished liver metabolome and microbiome function. Gas chromatography assessment of hepatic (A) UFA mol% and (B) ω6/ω3 ratios. (C) Glycerophospholipid patterns in GP1 (positively correlated with hepatic steatosis) and GP2 (negatively correlated with hepatic steatosis) WGCNA metabolomic modules. Metabolites were annotated with MassTRIX: Mass TRanslator into Pathways v. 3 and reported in Table S3. (D) WGCNA correlation analyses (39) clustered untargeted metabolites in 52 highly correlated modules. Each cluster (module eigengene or ME) was assigned a random color (displayed to the left). The figure shows the correlation between module eigengene and differentially abundant fecal microbiome pathways (individual murine stool samples) determined by PICRUSt (only displaying *P*adj* *of <0.0002). Circle size and coloring represent Spearman correlation coefficient (significance = *P*adj* *<* *0.05) between metabolomic module and clinical/group traits. A dagger indicates annotated modules in Table S3. See Text S1 for detailed methodology. The panel contains data from reversal profiling within the same experiment (*n *=* *8 per group). Bars indicate mean and SEM with statistical significance determined by one-way ANOVA with *posthoc* Tukey’s test; in panel A, asterisks indicate significance as reported in the legend to Fig. 4. Download FIG S6, TIF file, 2.7 MB. Copyright © 2020 Bauer et al. 2020 Bauer et al. This content is distributed under the terms of the Creative Commons Attribution 4.0 International license.

10.1128/mSystems.00499-20.1 TEXT S1 R code markdown of microbiome 16S rRNA and metabolomics analyses (WGCNA). Download Text S1, PDF file, 0.4 MB. Copyright © 2020 Bauer et al. 2020 Bauer et al. This content is distributed under the terms of the Creative Commons Attribution 4.0 International license.

10.1128/mSystems.00499-20.10 TABLE S3 WGCNA module data set. Download Table S3, CSV file, 0.06 MB. Copyright © 2020 Bauer et al. 2020 Bauer et al. This content is distributed under the terms of the Creative Commons Attribution 4.0 International license.

While PCA and metabolite set enrichment analysis revealed diet-induced alterations of phenylacetate, retinol, and fatty acid metabolism, the critical metabolites specifically linked to hepatic steatosis remained uncertain. Finally, we also sought to explore whether these metabolic shifts were associated with, or uncoupled from, microbiome features.

To address these unknowns, we conducted undirected, weighted gene coexpression network analysis (WGCNA) with untargeted metabolomic data. Using the WGCNA R package (39), highly correlated metabolites were clustered into 52 modules across samples without foreknowledge of metabolomic function. Module relationship to clinical traits, including hepatic histology and triglyceride content, the definitive diagnostic features of NAFLD (4, 8), was determined by Spearman rank correlation (*P*adj* *<* *0.05). Two modules significantly correlated with fatty liver traits: the “yellow” module (positive correlation) and “turquoise” module (negative correlation). These modules also correlated with the end diet (reversal diet), but not starting diet, indicating that metabolites within these modules were responsive to dietary intervention (Fig. 8C). Metabolites within these modules and the “red” module, which was not correlated with any group or clinical trait, were selected for further study and annotation.

Significantly correlated modules were predominantly comprised of glycerophospholipids, hereafter referred to as GP1 (for glycerophospholipid 1; yellow) (105 annotated, 183 nonannotated metabolites) and GP2 (for glycerophospholipid 2; turquoise) (243 annotated, 418 nonannotated). In contrast, the red module (28 annotated, 56 nonannotated metabolites) was largely comprised of cholanoic and taurocholic bile acid metabolites and was designated BA (Table S3). While GP1 and GP2 contain PE and phosphatidylcholine (PC) members, modules also exhibit distinct phospholipid patterns. The total number and relative abundance of glycerophosphoglycerols were more prevalent in GP1, while GP2 was enriched with glycerophosphoserines (Fig. S6C and Table S3). In addition, GP2, but not GP1, contains SFAs. We also examined the relationship between these modules and predicted microbiome functionality. GP1 and GP2, but not BA, display divergent and significant correlations with key PICRUSt pathways (Fig. S6D). This multiomic perspective not only identifies glycerophospholipid metabolism and fatty acid metabolism as key pathways linked to hepatic steatosis but also supports a causal role for the gut microbiota in driving undernutrition-induced fatty liver.

## DISCUSSION

While diet significantly influences NAFLD progression, NAFLD has largely been studied as a condition associated with overnutrition rather than undernutrition (7, 8). The MAL/MBG diet reflects dietary aberrations often observed during food insecurity—a poor diet comprised of refined carbohydrates and reduced intake of unsaturated fats and lean proteins (23, 40, 41). This form of malnutrition exists across both developed and developing countries (40, 42, 43). In addition, MBG mice model a “secondary hit” contributing to persistent undernutrition—a chronic exposure to fecal commensals due to poor sanitation/hygiene access and fecal-oral contamination (13, 44). MBG fecal contamination consists of E. coli and *Bacteroidales*, commensal microbes associated with both fatty liver and undernourished cohorts (21, 22, 36).

Although this model cannot capture the complex spectrum of malnutrition and the nonbiological forces driving health disparities in malnourished communities (e.g., socioeconomic status) (12), MBG pathology provides a unique opportunity to examine undernutrition-induced fatty liver. Here, we assessed the liver metabolome and gut microbiome, as altered metabolic and microbial pathways drive obese-associated NAFLD (36, 45). Further study of the undernourished gut-liver axis is needed to address additional molecular features (e.g., liver proteomic profiling) and/or go beyond presented analyses (e.g., metatranscriptomic studies to examine hypotheses generated from predictive PICRUSt data). Despite study limitations, MBG findings further knowledge of fatty liver within the context of dietary deficiency and gut microbial dysbiosis.

Prior studies have examined how undernutrition triggers fatty liver. In a protein-deficient rodent model, hepatic steatosis accompanied impaired mitochondrial fatty acid oxidation and hepatic peroxisome loss. Fenofibrate treatment, a peroxisome proliferator-activated receptor α (PPARα) stimulant, not only restored peroxisome deficits and improved mitochondrial function but also reduced hepatic steatosis, demonstrating a critical peroxisome-mitochondrion role in undernutrition-induced fatty liver (9). In addition, proinflammatory mediators, epigenetic modification, and reactive oxygen species (ROS) have also been implicated in the progression of pediatric malnutrition (2, 10). Fatty liver features have also been associated with intrauterine growth restriction and maternal undernutrition, further supporting a critical developmental window shaping liver health trajectories (10).

Beyond diet, the gut microbiome has also been implicated in the pathology of obese-associated fatty liver, particularly via modulation of bile acids (5, 19). Synthesized within the liver, bile acids are secreted in the small intestine. Gut microbes modify these primary bile acids, forming secondary bile acids (46). We have previously reported shifts in bile acid metabolism in our malnourished model, notably reduction of both primary and secondary tauro-conjugated bile acids, indicative of impaired host function and gut dysbiosis. In contrast, obese NAFLD/NASH cohorts exhibit increased plasma taurocholate levels (5, 47), perhaps suggestive of an overnourished NAFLD biomarker or systemic plasma profile. Surprisingly, undirected WGCNA following dietary intervention found no correlation between taurocholic acid module (BA) and fatty liver features, as well as between the BA module and key microbiome pathways. These findings suggest that alterations in taurocholic metabolism are potentially a consequence of, rather than contributor to, hepatic steatosis within the MBG and dietary reversal models.

Here, we report two hepatic metabolomic pathways linked to the MBG microbiome—phenylacetate and glycerophospholipid metabolism. While diet largely shaped the liver metabolome, hepatic steatosis was not observed following adult-onset malnutrition (C-MBG mice). To identify potential metabolomic distinctions between the MBG and C-MBG liver, we conducted metabolite set enrichment analysis which identified phenylacetic acid metabolism enriched in the MBG versus C-MBG metabolome. Phenylacetic acid was recently identified as a driver of hepatic steatosis in a cohort of obese, nondiabetic women (FLORINASH study). Researchers combined hepatic transcriptome, plasma/urine metabolomics, and fecal metagenomics to identify signatures and metabolic contributors of fatty liver. These multiomic analyses revealed disruption of AAAM, a bacterial pathway producing phenylacetic acid. Chronic phenylacetic acid exposure elevated hepatic triglyceride content, triggering NAFLD-like features in mice (36). PICRUSt predictions from our study also revealed elevated aromatic biosynthesis pathways in the malnourished microbiome prior to and following reversal treatment, supporting a microbiome-dependent role in undernutrition-induced NAFLD progression.

Both metabolite set enrichment analyses and WGCNA independently report aberrant lipid metabolism during undernutrition, notably altered glycerophospholipid and fatty acid metabolism. Altered glycerophospholipid profiles have been reported in murine and human cohorts of fatty liver disease and are implicated in hepatic steatosis pathology. For example, PE, a highly abundant mammalian glycerophospholipid, contributes to lipid signaling and serves as a precursor to PC. Both elevated PE metabolism and altered PC/PE glycerolipid ratios have been associated with fatty liver progression (28, 48–50). In murine livers, disruption of PE biosynthesis via disruption of the CDP-ethanolamine pathway triggered a 10-fold increase of triacylglycerol content in murine livers (28). While our methodology lacks the capacity to identify specific glycerophospholipid species driving hepatic steatosis, further study to explore glycerophospholipids as a mechanism driving hepatic steatosis and steatohepatitis is warranted.

Metabolite enrichment set analyses did highlight specific shifts in fatty acid profiles—PUFA metabolism. Key cell membrane components, PUFAs modulate inflammatory processes, lipid signaling, and triglyceride accumulation (29, 51, 52), with ω3 PUFA supplementation recently examined as a promising NAFLD treatment (29, 53). While MAL/MBG mice displayed a striking reduction of hepatic PUFA content, dietary intervention largely restored ω6 and ω3 PUFA profiles.

PUFAs are metabolized via fatty acid oxidation, a catabolic process influenced by vitamin A metabolites or retinoids. Storage and metabolism of retinoids largely occur within liver hepatocytes and hepatic stellate cells (37). Retinoids are key regulators of hepatic adiposity with retinaldehyde administration inhibiting diet-induced weight gain in mice (38, 54). Beyond fatty acid regulation, retinol, retinal, and retinoic acid contribute to diverse biological functions, including vision, adaptive T cell immunity, and gene transcription (55, 56). Prevalent in malnourished communities, vitamin A deficiencies drive vision impairments, growth deficits, and even mortality rates (57–59). Largely stored in the liver, hepatic steatosis is linked to vitamin A deficiencies (60). As both CON and MBG mice consume diets with equivalent vitamin A availability and exhibit comparable dietary retinol levels within the liver, retinal/retinoic acid deficits in malnourished mice likely reflect liver dysfunction (60). Like improved PUFA profiles, dietary intervention mitigated impaired vitamin A metabolism in MBG-R mice, most likely due to reduced hepatic steatosis. Clinical trials assessing vitamin A supplementation on anthropometric measurements in pediatric populations have reported promising, albeit inconsistent results (61, 62). These conflicting findings may result as a consequence of undernutrition-induced fatty liver and subsequent impairment of retinol metabolism.

**FIG 9:**
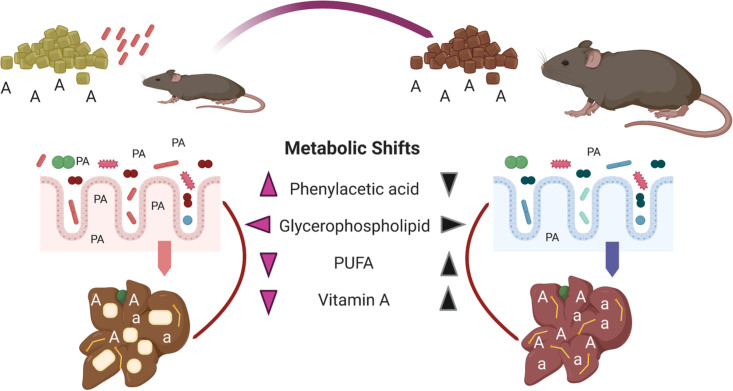
Multihit model of undernutrition-induced fatty liver and dietary intervention. Chronic exposure to specific, fecal microbes impacts hepatic steatosis and triglyceride accumulation in malnourished mice. Early-life malnutrition triggers an altered liver metabolome characterized by shifts in phenylacetate (phenylacetic acid [PA]), vitamin A (retinoids [A, a]), long-chain fatty acid, and glycerophospholipid metabolism. These changes are accompanied by striking alterations in gut microbiota community and function. Notably, enriched metabolism of phenylacetic acid, a bacterial product of AAAM metabolism, corresponds with increased relative frequency of AAAM predicted microbiome pathways in MBG mice, while altered glycerophospholipid metabolism correlates with both microbiome functional profiles and hepatic steatosis. Adult-onset malnutrition elicits metabolomic shifts largely uncoupled from hepatic steatosis (not shown), highlighting the importance of an early-life development period in liver function. In contrast, sustained dietary intervention largely mitigates microbial and host metabolic shifts during malnutrition, reducing hepatic steatosis and improving growth. Collectively, these findings demonstrate a putative role for commensal gut microbes in NAFLD and highlight putative host/microbial targets to reduce fatty liver burden in undernourished communities. This figure was created using Biorender.

In summary, we demonstrate that diet and the gut microbes alter multiple pathways that contribute to fatty liver features in a mouse model of early-life malnutrition. Malnutrition triggered diffuse macrovesicular lipidosis accompanied by (i) microbiome alterations and (ii) metabolomic shifts in phenylacetate, glycerophospholipid, PUFA, and vitamin A metabolism within the MBG liver. Beyond characterizing malnutrition-induced hepatic steatosis, our work highlights microbial-dependent shifts in composition and function which may contribute to fatty liver pathology and persistence. Sustained dietary intervention largely mitigated these aberrant features, while improving growth markers and reducing fatty liver histology (Fig. 9). In contrast, mice failed to exhibit diffuse macrovesicular lipidosis following adult-onset malnutrition and fecal-oral contamination, despite marked shifts in microbiome and metabolomic profiles. These alterations may precede fatty liver pathology in C-MBG mice maintained on a malnourished diet and/or may involve additional disruptions not captured in our study. Alternatively, our findings raise an intriguing possibility of a critical developmental window programming undernutrition-induced fatty liver within this model.

Childhood malnutrition and NAFLD remain global health concerns. The prevalence of malnutrition-induced fatty liver, specifically among pediatric populations, is expected to rise during the oncoming decades (2). Much research has examined NAFLD linked to one arm of the malnutrition spectrum—overnutrition and obesity. Our work provides a multifaceted assessment of undernutrition-induced fatty liver within an early-life model that addresses global health burdens, dietary deficiency, and gut microbiota dysbiosis (12, 13, 44). We anticipate that these findings will provide critical launching points to identify putative dietary, microbial, and/or metabolomic targets that address fatty liver pathology within undernourished communities.

## MATERIALS AND METHODS

### Mouse studies.

Newly-weaned female C57BL/6J mice were purchased from Jackson Laboratory and housed at the University of British Columbia Modified Barrier Facility (12-h light-dark cycle, *ad libitum* chow and water access). Mice were randomized into experimental groups with comparable starting weights and housed in ventilated cages filled with wood chip bedding (three to five mice per group). All mouse studies were approved by the Animal Care Committee at the University of British Columbia and the Canadian Council on Animal Care guidelines.

### MBG model.

Mice received either standard mouse chow “control diet” (catalog no. D09051102) or an isocaloric “malnourished diet” (catalog no. D14071001) developed by Research Diets, New Brunswick, NJ. A subset of mice on the malnourished diet were exposed to a cocktail of seven bacterial commensals (Bacteroides vulgatus 3/1/40A, Bacteroides fragilis 3/1/12, Bacteroides ovatus 3/8/47, Bacteroides dorei 5/1/36 [D4], Parabacteroides distasonis 2/1/33B, E. coli 3/2/53, and E. coli 4/1/47) given in a 1:1 ratio. Bacteria were plated in anaerobic conditions on fastidious anaerobe agar prior to oral gavage (100 μl). Following 2 weeks (week 5) on the control or malnourished diet, all mice received a series of three gavages administered every other day: MBG (10^9^ bacterial cells/ml in sterile, reduced phosphate-buffered saline [PBS]), non-MBG groups (sterile, reduced PBS). Full methodology and further dietary reports are provided in references 13 and 20. Upon sexual maturation, ∼6 weeks, mice were considered adults.

### Micro-CT.

Micro-CT scans were completed on 7-week-old anesthetized (isoflurane) mice within the Centre for High-Throughput Phenogenomics at the University of British Columbia using the eXplore CT 120 (TriFoil Imaging, Chatsworth, CA, USA). Micro-CT scanning was conducted with in-house protocols (rotation mode, continuous; single scan time, 4 min; entrance dose, 175 mGy). Image data sets were reconstructed into three-dimensional volumes (isotropic voxel size, 100 μm). On the basis of published methodology (63), we classified tissues into adipose, lean, and bone tissue with MicroView software (GE Healthcare Biosciences) with the following signal-intensity thresholds −200 to −275, −30 to −40, and 190 to 250 HU, respectively.

### Histology measurements.

Individual liver lobes were stored in 10% formalin for 12 to 24 h at room temperature. Following formalin storage, tissues were transferred into 70% ethanol. Paraffin-embedded tissues were sliced and stained with H&E using established practices by the Biomedical Research Centre (Ingrid Barta) or Wax-it Histology Services at the University of British Columbia. H&E tissues were imaged under a light microscope at 40×, and the percentage of fat- or fat/glycogen-associated per image was determined by Fiji (Image J) on 8-bit images. The threshold of “open” space was set by CON histology, and the same threshold settings were applied to all samples.

### *Ex vivo* cytokine quantification.

Liver tissues were collected for cytokine analysis. Tissue samples were stored in 1 ml of PBS with cOmplete EDTA-free protease inhibitor prior to homogenization and frozen at −70/−80°C. Tissue homogenates were centrifuged at top speed (≥16,000 × *g*) for 15 min at 4°C, and the resulting supernatants were stored at −80°C. Cytokine levels from liver supernatants were measured using the BD Biosciences cytometric bead array mouse inflammation kit. All cytokine concentrations were normalized to starting tissue weight.

### Triglyceride, glucose, and insulin.

Triglyceride measurements were determined from liver supernatants using the abcam triglyceride assay kit (ab65336). Triglyceride levels were first normalized to starting tissue weight and then compared against CON samples. Following euthanasia, blood was collected from nonfasted and fasted (overnight) mice. Immediately upon collection, blood glucose was measured via glucometer, while insulin levels were measured from mouse sera by ALPCO Mouse Insulin ELISA kit (catalog no. 80-INSMS-E10). Enzyme-linked immunosorbent assays (ELISAs) were completed according to the manufacturer’s recommendations.

### Untargeted metabolomics and metaboanalyst analyses.

Untargeted metabolomics (RP-UPLC-FTMS, HILIC-FTMS) were conducted by The Metabolomics Innovation Centre. Murine liver lobes were collected and weighed postmortem. Prior to analyses, tissues were kept in storage at −70/−80°C.

**(i) RP-UPLC-FTMS analysis.** Individual mouse liver tissues in 5 μl water/mg of liver tissue, plus two 4-mm metal balls were homogenized on a MM 400 mill (2×, 30 Hz for 1 min). Following a 5-s spin down, methanol-chloroform (4:1) at 25 μl/mg liver tissue was added to the Eppendorf tube. Following repeated homogenization method, samples were placed in an ice-water bath for sonication (5 min) and centrifuged for 20 min (15,000 rpm, 10°C). A 60-μl aliquot of supernatant from each sample was dried using the same nitrogen evaporator, and subsequent residue was reconstituted in 80% methanol (40 μl). Ten-microliter portions of these samples were used for reversed-phase RP-UPLC-FTMS. RP-UPLC-FTMS runs utilized the Waters BEH C_8_ (2.1 × 50 mm, 1.7 μm) column with 0.01% formic acid in water (mobile phase A) and 0.01% formic acid in 1:1 acetonitrile-isopropanol (mobile phase B). The mobile phase elution gradient was 5 min for 5% to 50% in mobile phase B, 15 min for 50% to 100% in mobile phase B, and 2 min in 100% mobile phase B. The column temperature was maintained at 60°C with a flow rate of 400 μl/min. Full RPLC-FTMS methodology is reported at the NIH Common Fund’s Data Repository and Coordinating Center website (studies ST001367 and ST001368).

**(ii) HILIC-FTMS analysis.** Individual sample supernatants were mixed with 120 μl of water, 180 μl of methanol, and 195 μl of chloroform. The mixture was vortex mixed at 3,000 rpm for 30 s before centrifugal clarification. Three hundred microliters of the upper, aqueous phase was precisely taken out and transferred to a “V”-shape LC injection microvial and dried down under a gentle nitrogen gas flow in the nitrogen evaporator. The residue was reconstituted in 50 μl of 80% acetonitrile. Ten microliters was injected for HILIC-FTMS.

HILIC-FTMS was performed on a Waters HILIC column (2.1 × 100 mm, 1.8 μm) for chromatographic separation of very polar metabolites. The mobile phase was as follows: mobile phase A was 0.01% formic acid in water, and mobile phase B was 0.01% formic acid in acetonitrile. For binary gradient elution, 85% mobile phase B for 1 min; 85% to 25% mobile phase B in 8 min, followed by column equilibration at 85% mobile phase B for 6 min between injections. The flow rate was 0.3 ml/min, and the column temperature was 30°C. The MS instrument was run in the survey scan mode with FTMS detection at a mass resolution of 60,000 full width at half maximum (FWHM) at *m/z* 400. Two HILIC-FTMS data sets were acquired for each sample, one with positive-ion detection and the other with negative-ion detection. The mass scan range was *m/z* 80 to 800.

**(iii) Data processing and analyses.** Data processing and analyses were conducted with XCMS (https://xcmsonline.scripps.edu/) in R to procure *m/z* (mass-to-charge ratio), retention time (RT) (in minutes), and LC-MS peak areas. To assign the metabolite candidates of any potential biomarkers, the measured *m/z*’s were searched against metabolome databases, namely, METLIN (Scripps Research Institute) with mass errors of ≤3 ppm. For positive ion detection data, (M+H)+, (M+Na)+, (M-H2O+H)+, and (M-NH3+H)+ were allowed in database searches. For negative ion detection, (M-H)-, (M+Na-2H)-, (M-H2O-H)-, and (M-NH3-H)- were allowed. PCA plots, enrichment analyses, and pathway analyses were carried out using Metaboanalyst v. 4.0 software: mass tolerance, 0.0003; retention time tolerance, 30; data filtering, nonparametric relative standard deviation (MAD/median), normalized from pooled CON samples, log transformation, and auto data scaling. A one-way ANOVA was used to determine significant changes between groups (*P*adj* *<* *0.05; fold change >2). Analyses were completed based on previously reported studies (13). All metabolite set enrichment analyses were conducted using small molecule pathway database (SMPDB), unless otherwise stated.

### Vitamin A metabolomics.

Vitamin A metabolites were assessed at The Metabolomics Innovation Centre. Mouse liver tissue was homogenized in 50% aqueous methanol (25 μl/mg tissue) in Eppendorf tubes with two 4-mm metal balls/tube using the MM 400 mixer mill (shaking frequency, 30 Hz for 1 min × 2), followed by sonication in a water bath for 2 min. Hexane (50 μl/mg tissue) containing 20 μg/ml butylated hydroxytoluene (BHT) (antioxidant) was added to the tube. The mixture was vortex mixed at 3,000 rpm for 30 s before 6-min centrifugation at 15,000 rpm, and at 10°C, the whole phase was split into an upper organic phase and a lower aqueous phase. The organic phase was removed with a gel-loading tip, and the aqueous phase was extracted with hexane again at 50 μl/mg tissue. After centrifugation, the organic-phase extracts from two rounds of liquid-liquid extraction were combined and then dried in a nitrogen evaporator. The residue was dissolved in methanol (5 μl per mg tissue), containing 0.5 μg/ml of beta-tocopherol-D3 as internal standard. 10 μl of sample was injected to a C_8_ UPLC column (2.1 × 50 mm, 1.7 μm) to run UPLC-high-resolution MS on a Thermo Scientific LTQ-Orbitrap mass spectrometer, which was operated with positive-ion FTMS detection at 60,000 FWHM (*m/z* 400) in a mass scan range of *m/z* 100 to 1800. Serially diluted, mixed standard solutions of fat-soluble vitamin A (retinol, retinal, and retinoid acid) in a concentration range of 0.01 to 100 nmol/ml per compound were prepared in the same internal standard solution, and 10-μl aliquots were injected to acquire the data to construct the linear calibration curves for the quantitation. The mobile phases were a 5 μM silver-ion solution (mobile phase A) and acetonitrile-isopropanol (1:1) (mobile phase B) for binary-solvent gradient elution, with a gradient of 30% to 100% mobile phase B in 10 min at a flow rate of 250 μl/min. Vitamin A concentrations were calculated from the linear regression calibration curves of their standard compounds.

### Liver fatty acid profile.

Liver tissues were collected and immediately placed in dry ice prior to storage at −70/−80°C prior to processing. Liver tissues (50 mg × sample) were homogenized at 4°C with an Ultra-Turrax homogenizer (IKA, Staufen, Germany) in homogenization buffer (1 μM 2,6-di-*tert*-butyl-4-methylphenol, 1 mM diethylenetriamine penta-acetic acid, 2 mM ethylenediamine tetra-acetic acid, 5 mM 3-(*N*-morpholino)propanesulfonic acid, with 180 mM potassium chloride, and adjusted to pH 7.4). Samples were normalized by protein content (Bradford assay). Tissue fatty acid profiles were based on reported methodology (64).

**(i) Fatty acid methyl ester preparation.** Total lipids from liver homogenates were extracted via chloroform-methanol (2:1 [vol/vol]; 3 times) with 0.01% butylated hydroxytoluene. The chloroform phase was evaporated under nitrogen, and the fatty acids were transesterified by incubation in 2.5 ml of 5% methanolic HCl at 75°C for 90 min. Following transesterification, 2.5 ml of *n*-pentane and 1 ml of saturated NaCl solution were added to extract fatty acid methyl esters (FAMEs). The *n*-pentane phase was separated, evaporated under N_2_ gas, and redissolved in 80 μl of carbon disulfide. Two microliters was used for subsequent GC analysis.

**(ii) Gas chromatography conditions.** Gas chromatography analyses was performed on a GC system 7890A with a series injector 7683B and a flame ionization detector (Agilent Technologies, Barcelona, Spain), equipped with a DBWAX capillary column (length of 30 m, inner diameter of 0.25 mm, and film thickness of 0.20 μm). The injection port was maintained at 220°C and the detector at 250°C. Injections were performed using the splitless mode. The flow rate of carrier gas (helium 99.99%) was maintained at 1.8 ml/min. The temperature program was 5 min at 145°C, then 2°C/min to 245°C, and finally, the temperature was held at 245°C for 10 min with a postrun of 250°C for 10 min.

**(iii) Data analysis.** Identification of FAMEs was made by comparison with authentic standards (Larodan Fine Chemicals, Malmö, Sweden). The fatty acid profile detected plus identified plus quantified represents more than 95% of the total chromatogram. Results were expressed as percent moles. Here, we normalized against pooled CON samples.

### Microbiome and multiomic analyses.

**(i) 16S sequencing and analyses.** Collected fecal pellets were stored in −70°C prior to DNA isolation with the QIAamp PowerFecal DNA kit (Qiagen catalog no. 12830). Library preparation for 16S rRNA sequencing was performed with barcoded primers (V4 region) as described in reference 65. Upon ensuring successful amplification via gel electrophoresis, PCR amplicons were cleaned and normalized with the Sequal-Prep kit (ThermoFisher catalog no. A1051001), pooled, and sequenced on an Illumina MiSeq (v2 kit, 2 × 250 bp reads).

Demultiplexed reads were analyzed and annotated in QIIME2 (v 2018.2) using the DADA2 pipeline (sampling depth of 22051 bp) and Greengenes 97% operational taxonomic units (OTUs) (66–68). Additional filtering excluded contaminants (mitochondria, chloroplast). QIIME provided *P*adj for bacterial families. Downstream microbiome analyses and visualization were performed in R; further details are provided in Text S1 in the supplemental material.

**(ii) PICRUSt.** To assess functional changes in the fecal microbiota, we conducted PICRUSt (v2.1.3b). The full output is available in Table S2. Metabolic pathways were annotated using MetaCyc (35, 69).

**(iii) WGCNA.** WGCNA R package (39) identified metabolomic modules (modular eigengene) that correlated with both clinical features, traits, and PICRUSt output (Spearman rank correlation test, *P*adj* *<* *0.05). WGCNA was completed on the less polar metabolomic data (positive run) following normalization (as described above in “Untargeted metabolomics and metaboanalyst analyses”). On the basis of a scale-free topology, we chose a soft threshold β  = 13. Modules containing ≥5 metabolites were identified, and full clustering criteria and the R code are given in Text S1. Modules were auto-labeled by color. Metabolites within modules of interest were auto-annotated using MassTRIX: Mass TRanslator into Pathways v. 3 (70) (Table S3).

### Statistical analysis.

Statistical analyses provided were performed with GraphPad Prism software version 7.00/8.00. Results are expressed as the means with standard error of the means (SEM), unless otherwise stated.

### Data availability.

The data sets generated and/or analyzed during the current study are available from the corresponding author upon request. Microbiome and WGCNA pipelines are reported in Text S1. Raw metabolomic data have been deposited at the NIH Common Fund’s National Metabolomics Data Repository (NMDR) website, the Metabolomics Workbench (https://www.metabolomicsworkbench.org), where it has been assigned study identifier (ID) PR000935. These data can be accessed directly via https://doi.org/10.21228/M8TT3R. NMDR is supported by NIH grant U2C-DK119886. Raw 16S sequencing has been deposited in the NCBI Sequence Read Archive (SRA) under accession number PRJNA629327.
