# Supplementary figures and images for: Dietary Intervention Reverses Fatty Liver and Altered Gut Microbiota during Early-Life Undernutrition
Source: mSystems. 2020 Sep 8;5(5):e00499-20. doi: 10.1128/mSystems.00499-20 (PMC7483509; doi:10.1128/mSystems.00499-20)

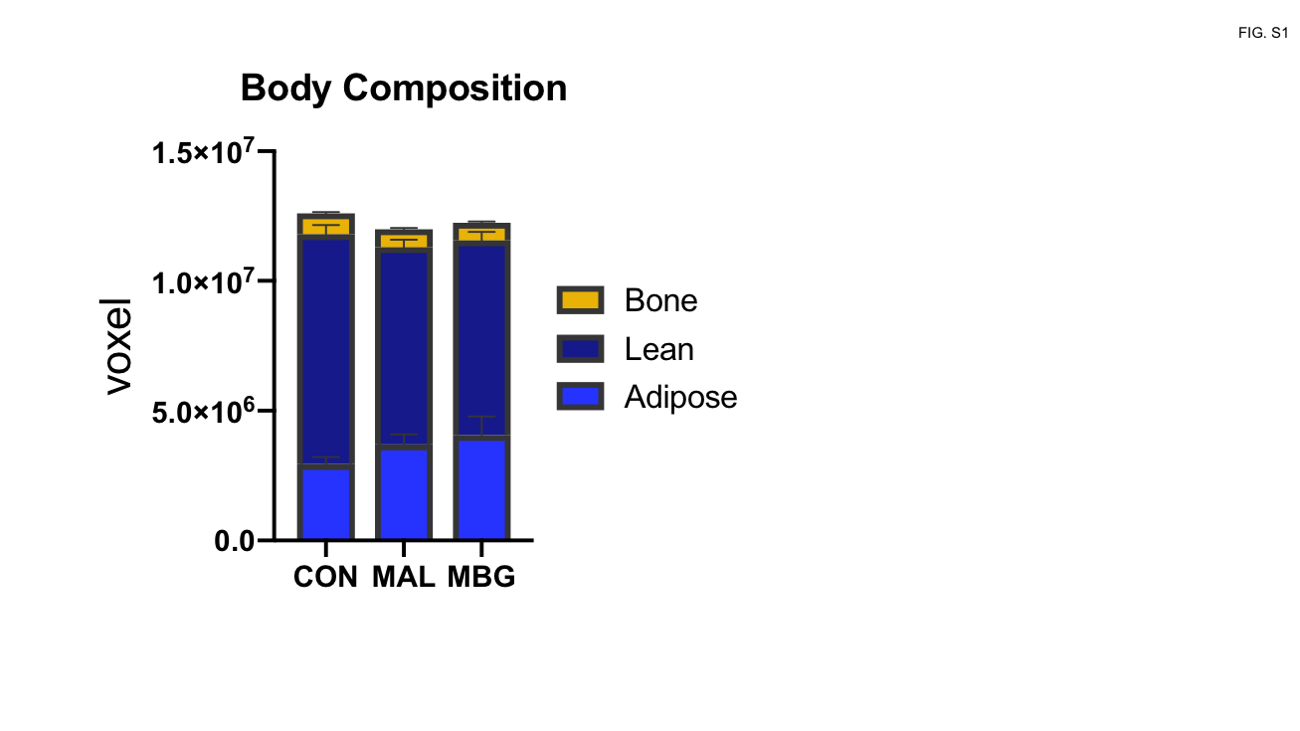

Supplement: FIG S1 [file mSystems.00499-20-sf001.tif]

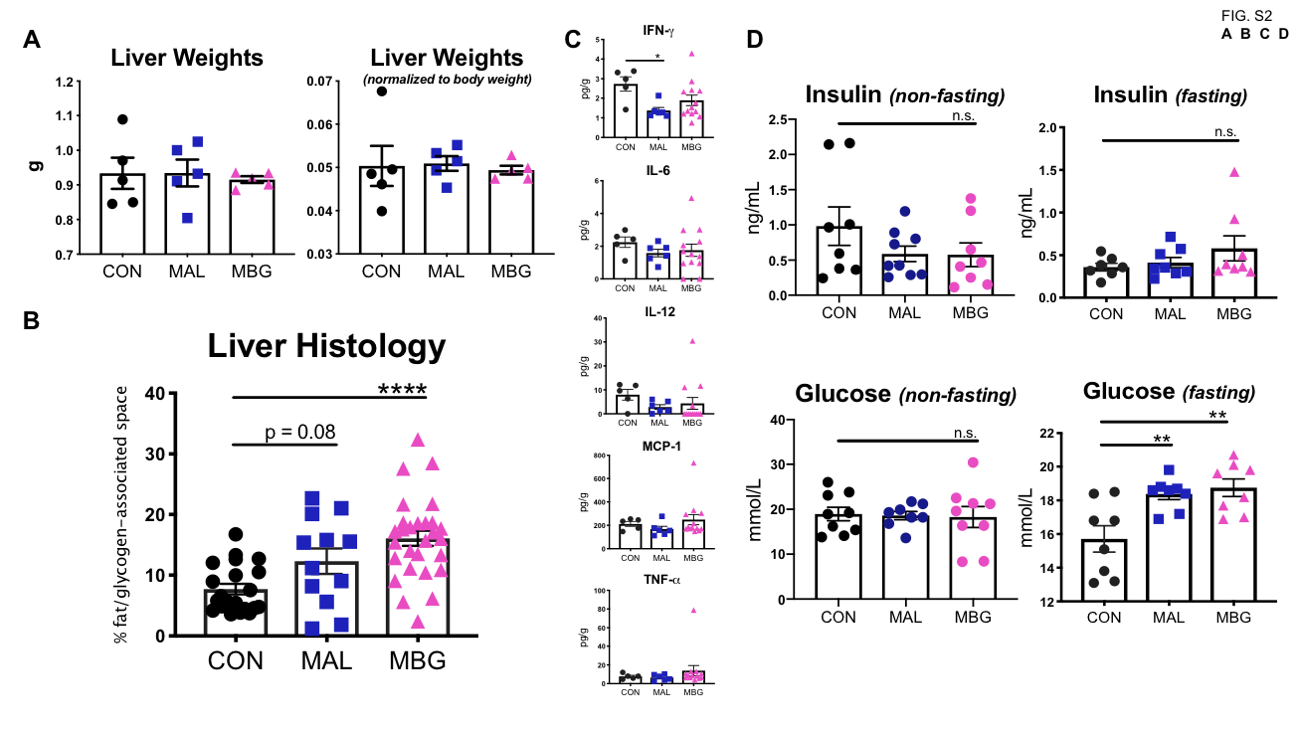

Supplement: FIG S2 [file mSystems.00499-20-sf002.tif]

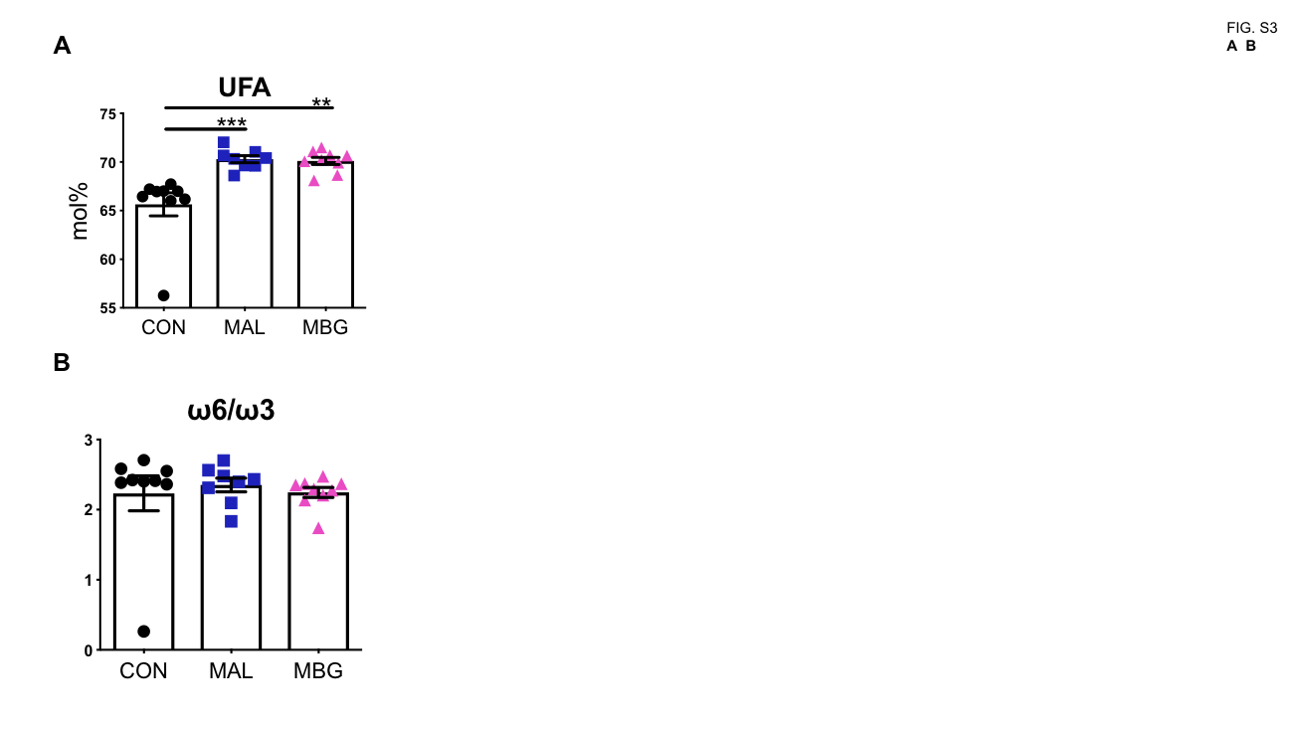

Supplement: FIG S3 [file mSystems.00499-20-sf003.tif]

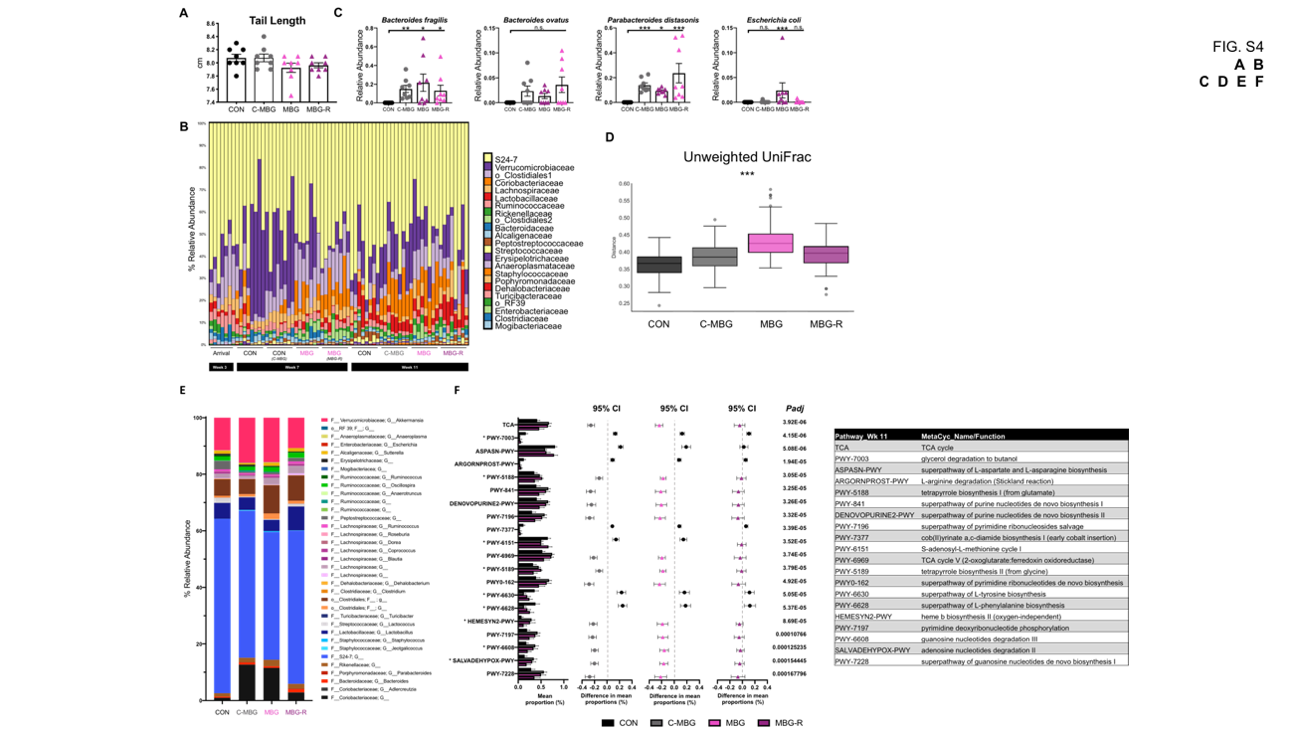

Supplement: FIG S4 [file mSystems.00499-20-sf004.tif]

## Slide 1
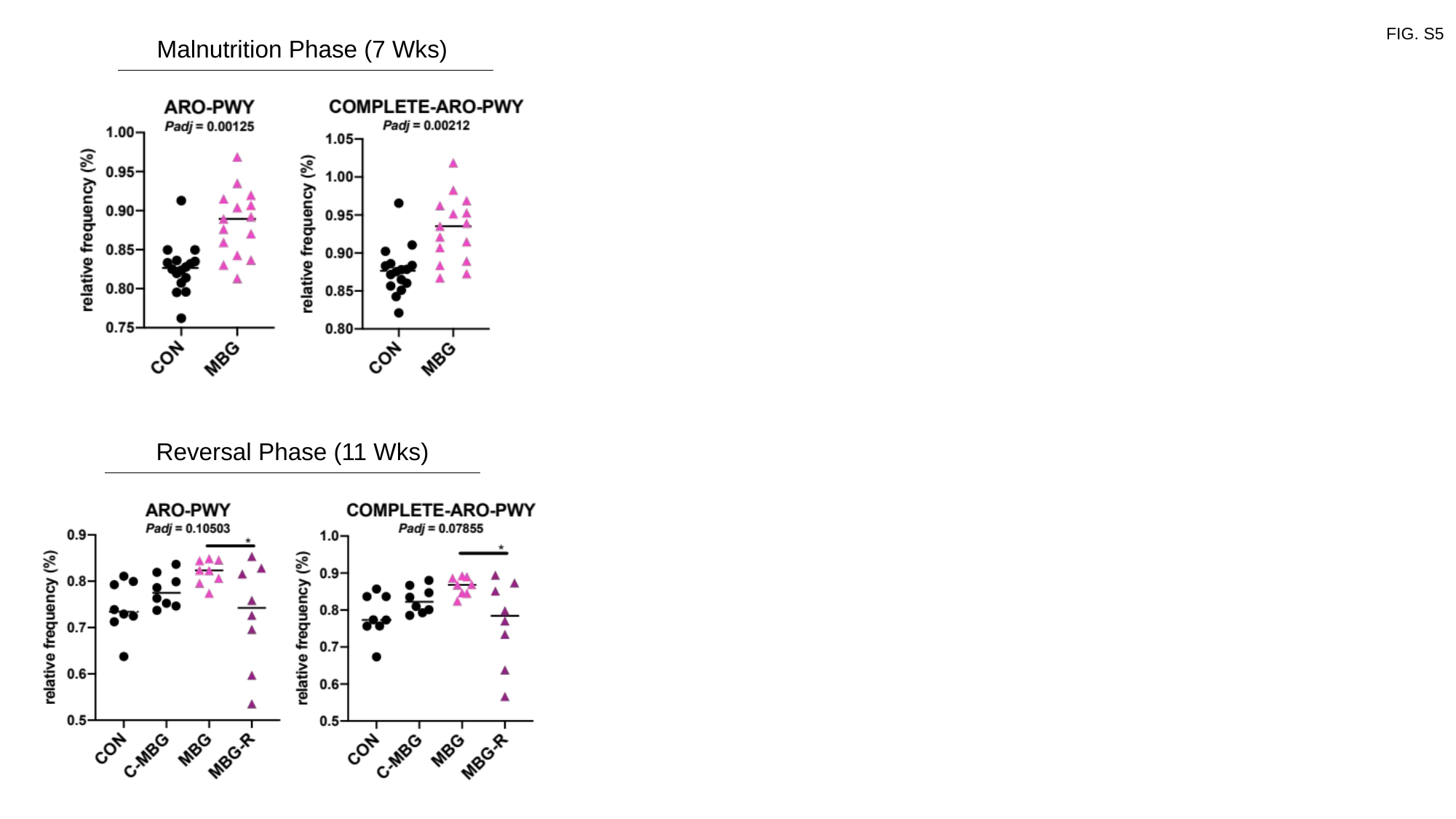

FIG. S5
Malnutrition Phase (7 Wks)
Reversal Phase (11 Wks)

Supplement: FIG S5 [file mSystems.00499-20-sf005.pptx]

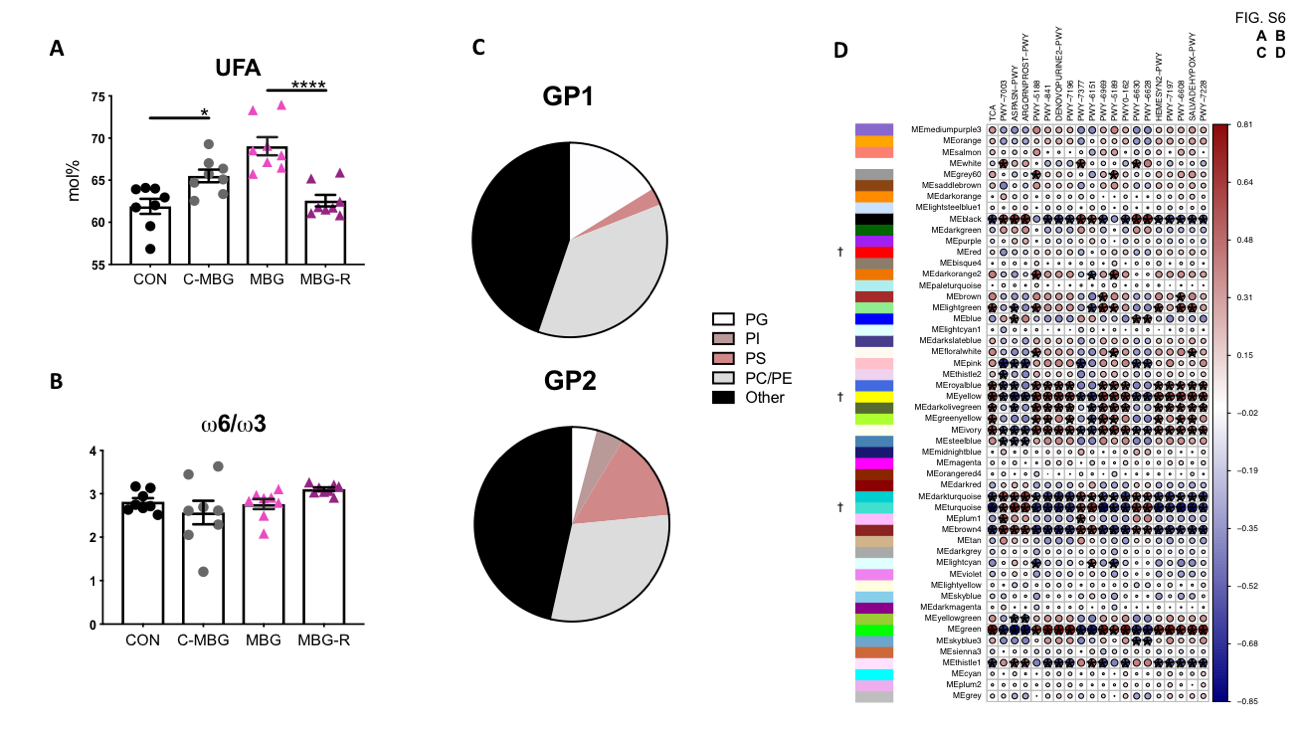

Supplement: FIG S6 [file mSystems.00499-20-sf006.tif]
