# Supplementary material for: Dietary Intervention Reverses Fatty Liver and Altered Gut Microbiota during Early-Life Undernutrition
Source: mSystems. 2020 Sep 8;5(5):e00499-20. doi: 10.1128/mSystems.00499-20 (PMC7483509; doi:10.1128/mSystems.00499-20)
Supplement: TEXT S1 [file mSystems.00499-20-s0001.pdf]

Microbiome_Metabolomics

Kylynda C. Bauer

2020-04-03

Contents

RCode_MICROBIOME_WGCNA **1**
Microbiome_Alpha_Beta_Tests 2
WGCNA 4

```
library(knitr)
```

```
## Warning: package 'knitr' was built under R version 3.5.2
```

```
opts_chunk$set(tidy.opts=list(width.cutoff=60),tidy=TRUE)
```

RCode_MICROBIOME_WGCNA

RPackages_Set Working Directory

```
library(corrplot)
library(dplyr)
library(phyloseq)
library(psych)
library(qiime2R)
library(tibble)
library(tidyr)
library(tidyverse)
library(WGCNA)
#### Important to add..
options(stringsAsFactors = FALSE)
allowWGCNAThreads()
```

Pipeline Sources

MICROBIOME:

https://forum.qiime2.org/t/tutorial-integrating-qiime2-and-r-for-data-visualization-and-analysis-using-qiime2r/4121/19

WGCNA:

https://horvath.genetics.ucla.edu/html/CoexpressionNetwork/Rpackages/WGCNA/Tutorials/

Microbiome_Alpha_Beta_Tests

Mouse Age Week 11 (Week 8 of experiment) reported here, Week 7 analyses performed with identical code, but Week 7-specific files

Data Input from Microbiome Analyses

```
# Bring in Metadata
metadata <- read_tsv("Metadata8_NAMES.txt")
```

```
## Parsed with column specification:
## cols(
##   `#SampleID` = col_character(),
##   Time_Week = col_double(),
##   Group = col_character(),
##   `Starting Diet` = col_character(),
##   Ending_Diet = col_character(),
##   Cage = col_character()
## )
```

```
metadata

# Bring in table data from QIIME2 analyses
SVs <- read_qza("8-filtered-table.qza")
names(SVs)  #information about the object
SVs$uuid
SVs$data[1:5, 1:5]

# Bring in taxonomy from QIIME2 analyses
taxonomy <- read_qza("taxonomy.qza")
# convert the table into a tabular split version
taxtable <- taxonomy$data %>% as.tibble() %>% separate(Taxon,
    sep = "; ", c("Kingdom", "Phylum", "Class", "Order", "Family",
        "Genus", "Species"))
```

```
## Warning: `as.tibble()` is deprecated, use `as_tibble()` (but mind the new semantics).
## This warning is displayed once per session.
```

```
## Warning: Expected 7 pieces. Missing pieces filled with `NA` in 713 rows [11, 18,
## 32, 33, 38, 42, 49, 55, 56, 59, 66, 69, 71, 73, 77, 81, 83, 84, 90, 99, ...].
```

```
head(taxtable)

# Bring in rooted tree from QIMME2 analyses
```

```r
tree <- read_qza("rooted-tree.qza")
tree$uuid
tree$data

### Alpha Diversity_Beta Diversity from QIMME2 analyses
### (additional analyses can be completed e.g.
### Shannon/Bray-Curtis etc.)
faithpd <- read_qza("faith_pd_vector.qza")
faithpd$uuid

pco1w8 <- read_qza("unweighted_unifrac_pcoa_results.qza")
pco1w8$uuid
head(pco1w8$data$ProportionExplained)
pco1w8$data$Vectors[1:5, 1:3]
```

Visualization for Microbiome Analyses

```r
UUPlay1w8 <-pco1w8$data$Vectors %>%
  rename("#SampleID"=SampleID) %>% #rename to match the metadata table
  left_join(metadata) %>%
  left_join(faithpd$data %>% rownames_to_column("#SampleID")) %>%
  ggplot(aes(x=PC1, y=PC2, color=Group, size=faith_pd, shape=Ending_Diet)) +
  geom_point() +
  xlab(paste("Principal Component 1:", round(100*pco1w8$data$ProportionExplained[1],
                                             digits = 1), "%")) +
  ylab(paste("Principal Component 2:", round(100*pco1w8$data$ProportionExplained[2],
                                             digits = 1), "%")) +
  theme_bw() +
  ggtitle("Unweighted UniFrac")
```

```
## Joining, by = "#SampleID"
## Joining, by = "#SampleID"
```

```r
UUPlay1w8 + scale_size_continuous(range=c(4,8)) +
  scale_color_manual(limits = c("CON", "C-MBG","MBG", "MBG-R"),
                     values=c("black","grey","deeppink3", "deeppink4")) +
  theme(title = element_text(size=18, face = "bold"),
        axis.title.x = element_text(size = 16),
        axis.title.y = element_text(size = 16), legend.text = element_text(size = 16)) +
  guides(colour = guide_legend(override.aes = list(size=5))) +
  guides(shape = guide_legend(override.aes = list(size=5)))
```

```r
#Further Aesthetics Code
UUPlay2w8 <-UUPlay1w8 + scale_size_continuous(range=c(4,8)) +
  scale_color_manual(limits = c("CON", "C-MBG","MBG", "MBG-R"),
                     values=c("black","grey","deeppink3", "deeppink4")) +
  theme(title = element_text(size=18, face = "bold"),
        axis.title.x = element_text(size = 16),
        axis.title.y = element_text(size = 16), legend.text = element_text(size = 16)) +
  guides(colour = guide_legend(override.aes = list(size=5))) +
  guides(shape = guide_legend(override.aes = list(size=5)))
```

```
UUPlay2w8
```

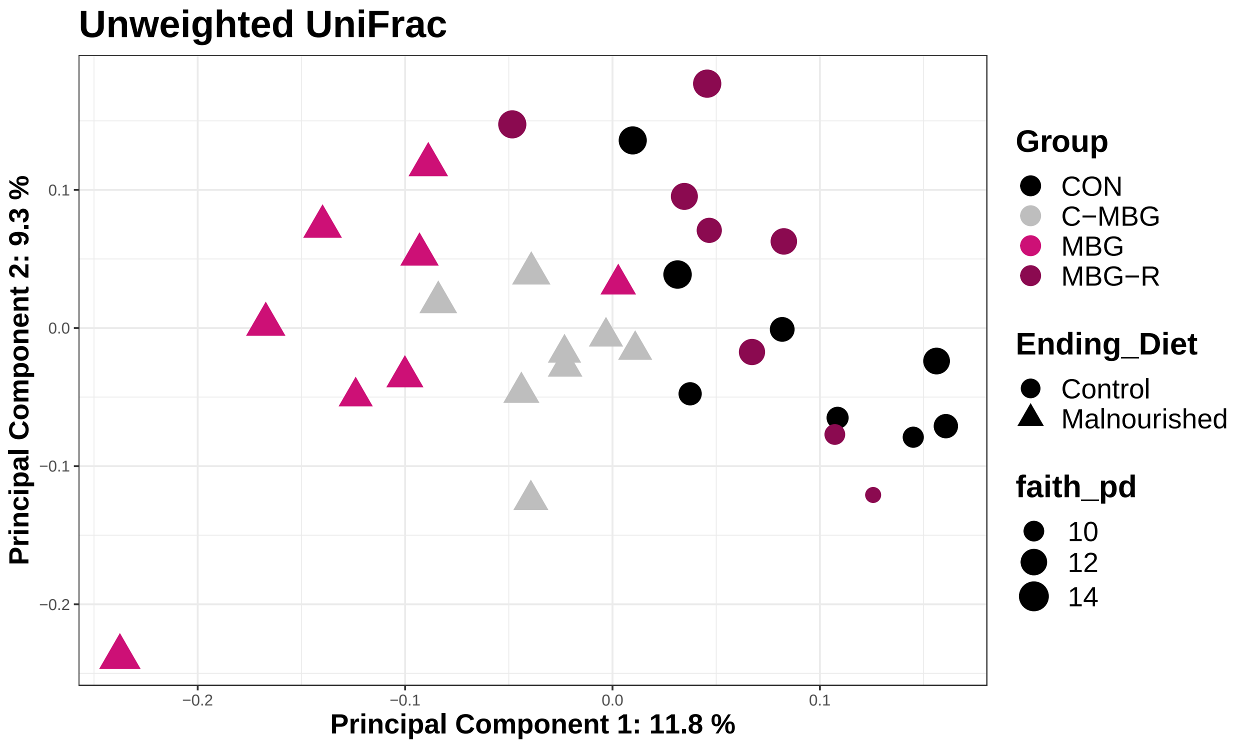

WGCNA

Showing correlation for clinical/group traits, you can change input depending on correlation (e.g. same code different input for PICRUSt)

Metabolomics Input Data

```
# Import normalized data from
# metaboAnalyst_Liver_less-polar_positive / Transpose_samples
# in rows and metabolites in columns
Metdata <- read.csv("normalizedMetabolomics43.csv", check.names = FALSE)
Metdata <- data.frame(Metdata[, -1], row.names = Metdata[, 1],
    check.names = FALSE)
Metdata <- data.frame(t(Metdata), check.names = FALSE)
head(Metdata)
dim(Metdata)
is.data.frame(Metdata)

# Checking that the data frame contains only numeric values.
sapply(Metdata, is.numeric)
Metdata <- data.frame(sapply(Metdata, as.numeric), check.names = FALSE,
    row.names = rownames(Metdata))
```

```r
# Checking for missing values, if TRUE no missing data
gsg = goodSamplesGenes(Metdata, verbose = 3)
```

```
##  Flagging genes and samples with too many missing values...
##   ..step 1
```

```r
gsg$allOK
```

```
## [1] TRUE
```

Check for Outliers

All samples were kept based on sampleTree and earlier Metabolomics PCA (in text)

```r
sampleTree = hclust(dist(Metdata), method = "average")
par(cex = 0.6)
par(mar = c(0, 4, 2, 0))
plot(sampleTree, main = "Sample clustering to detect outliers",
    sub = "", xlab = "", cex.lab = 1.5, cex.axis = 1.5, cex.main = 2)
```

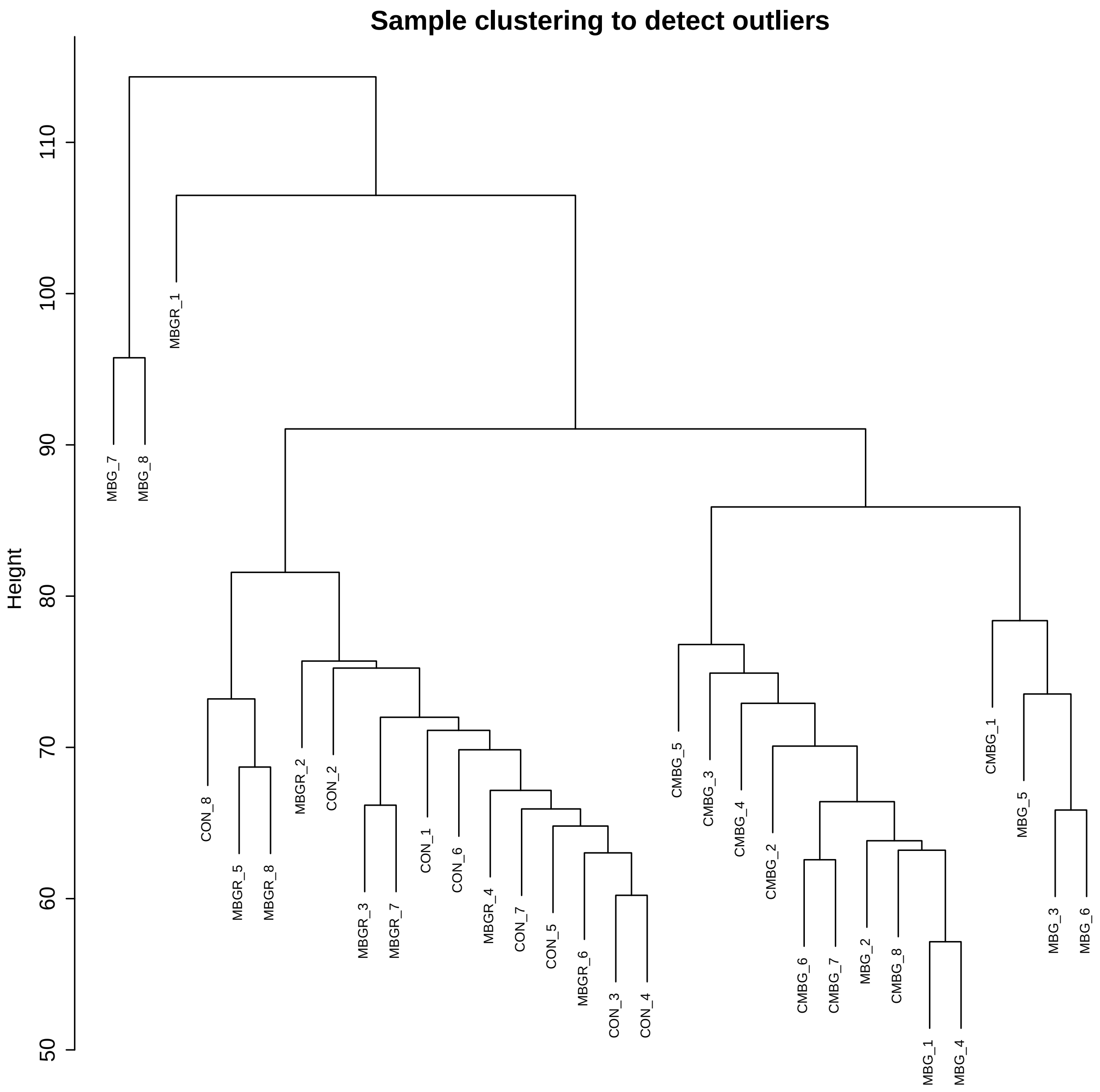

Clinical/Group (Binarize) Input

```
# Loading clinical trait data
MetTraits = read.csv("Metadata_M.csv", check.names = FALSE)
head(MetTraits)
MetTraits <- data.frame(MetTraits, check.names = FALSE)

# rder groups and binarize (Healthy, Malnourished), to make
# pairwise analyses
MetTraits$Groups = factor(MetTraits$Groups, levels = c("CON",
    "C-MBG", "MBG", "MBG-R"))
MetTraits$`Start Diet` = factor(MetTraits$`Start Diet`, levels = c("Healthy",
```

```
    "Malnourished"))
MetTraits$`Final Diet` = factor(MetTraits$`Final Diet`, levels = c("Healthy",
    "Malnourished"))

bin1 = binarizeCategoricalColumns(MetTraits$Groups, includePairwise = TRUE,
    includeLevelVsAll = FALSE)
bin2 = binarizeCategoricalColumns(MetTraits$`Start Diet`, includePairwise = TRUE,
    includeLevelVsAll = FALSE)
bin3 = binarizeCategoricalColumns(MetTraits$`Final Diet`, includePairwise = TRUE,
    includeLevelVsAll = FALSE)

# creating new trait data file for group traits
groupTraits <- data.frame(MetTraits$Sample_ID, bin1$`data.C-MBG.vs.CON`,
    bin1$data.MBG.vs.CON, bin1$`data.MBG-R.vs.CON`, bin1$`data.MBG-R.vs.MBG`,
    bin1$`data.MBG.vs.C-MBG`, bin2$data.Malnourished.vs.Healthy,
    bin3$data.Malnourished.vs.Healthy)
names(groupTraits) <- c("Sample_ID", "C-MBGvCON", "MBGvCON",
    "MBG-RvCON", "MBG-RvMBG", "MBGvC-MBG", "Start_Mal_Diet",
    "End_Mal_Diet")
groupTraits <- column_to_rownames(groupTraits, "Sample_ID")
head(groupTraits)

# Now for the non-binarized, continuous data
dataTraits = read.csv("Metadata_M.csv", check.names = FALSE)
dataTraits1 <- data.frame(dataTraits[, -1], row.names = dataTraits[,
    1], check.names = FALSE)
dataTraits2 <- dataTraits[, -c(1:5)]
head(dataTraits2)
```

```
# Sample dendrogram tree with group trait (binarized table)
sampleTree2 = hclust(dist(Metdata), method = "average")
# Convert traits to a color representation: white means low,
# red means high, grey means missing entry
traitColors = numbers2colors(groupTraits, signed = FALSE)
# Plot the sample dendrogram and the colors underneath.
plotDendroAndColors(sampleTree2, traitColors, groupLabels = names(groupTraits),
    main = "Sample dendrogram and group heatmap")
```

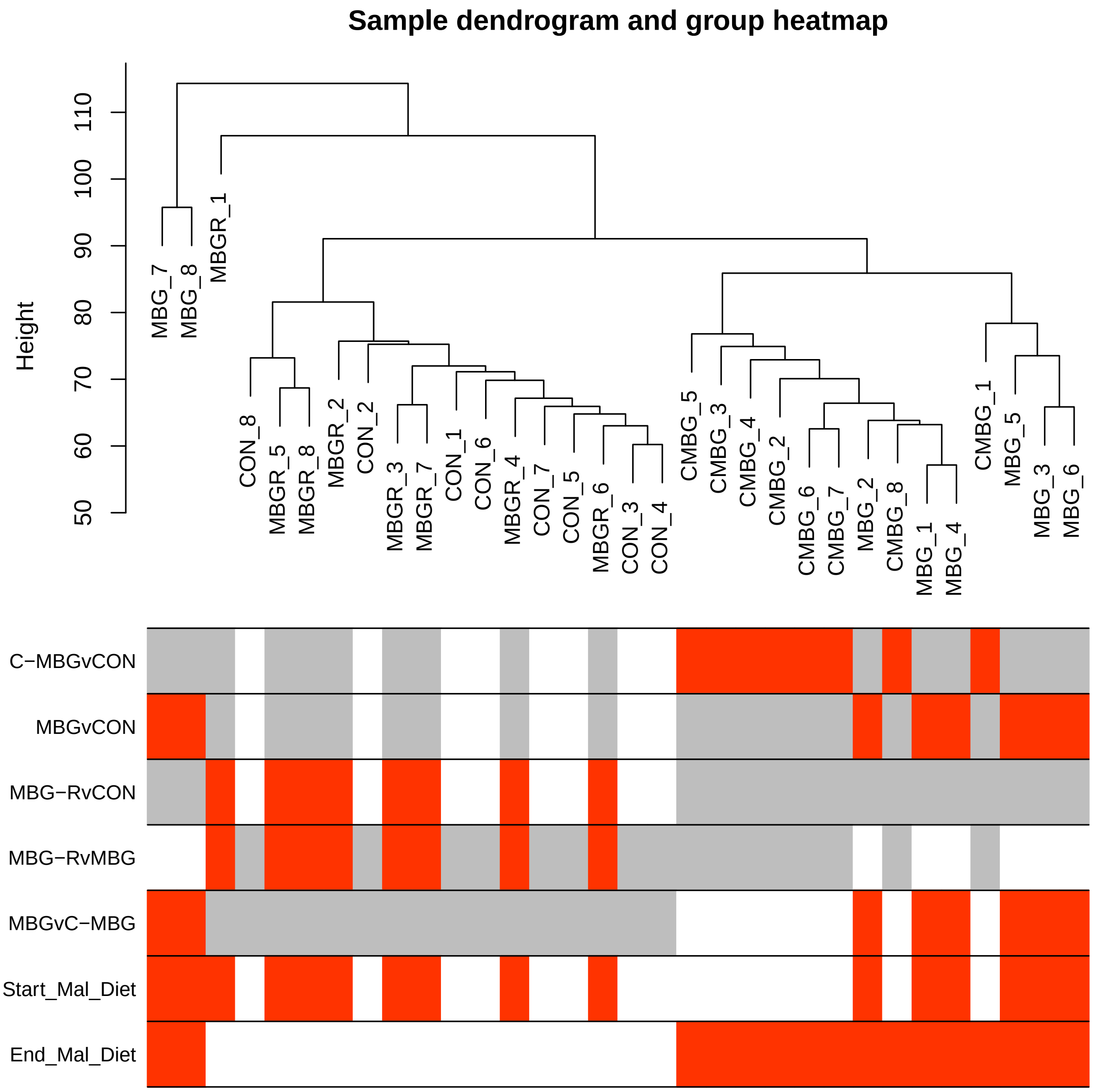


```
# Sample dendrogram tree with clinical traits (continuous)
sampleTree3 = hclust(dist(Metdata), method = "average")
# Convert traits to a color representation: white means low,
# red means high, grey means missing entry
traitColors = numbers2colors(dataTraits2, signed = FALSE)
# Plot the sample dendrogram and the colors underneath.
plotDendroAndColors(sampleTree3, traitColors, groupLabels = names(dataTraits2),
    main = "Sample dendrogram and clinical trait heatmap")
```

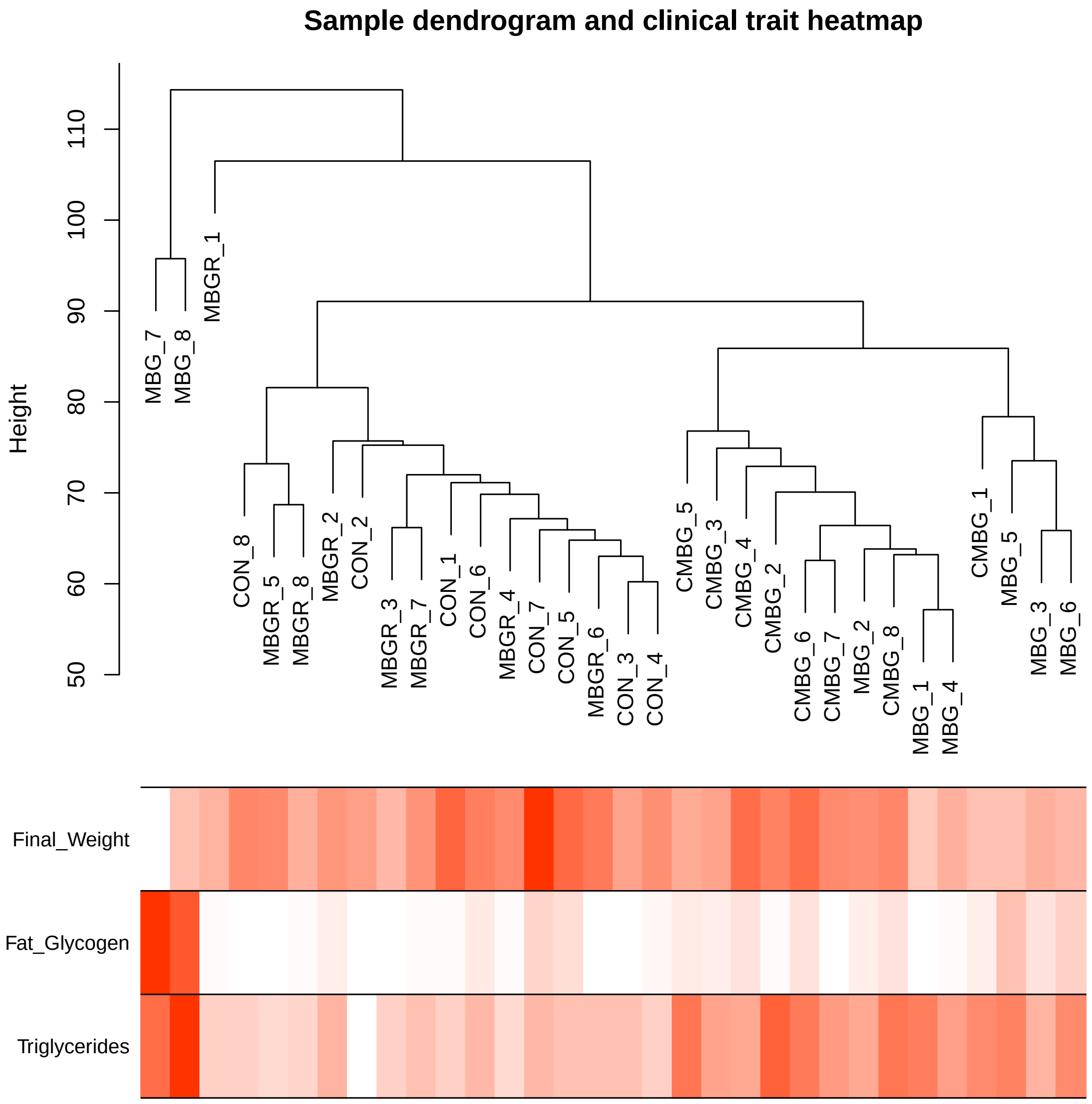

Forming Network Clusters

```
## Picking soft threshold, using WGCNA tutorial power settings
powers = c(c(1:10), seq(from = 12, to = 20, by = 2))
sft = pickSoftThreshold(Metdata, powerVector = powers, verbose = 5)
```

```
## pickSoftThreshold: will use block size 4068.
##  pickSoftThreshold: calculating connectivity for given powers...
##    ..working on genes 1 through 4068 of 4068
##    Power SFT.R.sq   slope truncated.R.sq mean.k. median.k. max.k.
## 1      1  0.00129 -0.0693          0.572  909.00   903.000 1460.0
## 2      2  0.47900 -0.9520          0.705  329.00   298.000  762.0
```

```
## 3      3  0.65500 -1.2100          0.796  153.00   119.000  474.0
## 4      4  0.70700 -1.3300          0.840   82.60    54.400  326.0
## 5      5  0.74000 -1.3700          0.881   49.60    28.300  237.0
## 6      6  0.75900 -1.4000          0.908   32.10    15.700  179.0
## 7      7  0.76600 -1.4300          0.920   21.90     9.130  138.0
## 8      8  0.78600 -1.4300          0.936   15.70     5.670  109.0
## 9      9  0.79500 -1.4300          0.942   11.60     3.710   88.0
## 10    10  0.80900 -1.4100          0.951    8.81     2.480   71.8
## 11    12  0.83700 -1.4100          0.961    5.48     1.290   50.6
## 12    14  0.84000 -1.3900          0.953    3.68     0.719   37.0
## 13    16  0.88000 -1.3100          0.960    2.62     0.395   27.9
## 14    18  0.94800 -1.2500          0.996    1.95     0.234   22.4
## 15    20  0.93300 -1.3600          0.994    1.52     0.144   21.1
```

```r
# Plot Scale Independence and Mean Connectivity
par(mfrow = c(1, 2))
cex1 = 0.9
### Scale-free topology fit index as a function of the
### soft-thresholding power
plot(sft$fitIndices[, 1], -sign(sft$fitIndices[, 3]) * sft$fitIndices[,
    2], xlab = "Soft Threshold (power)", ylab = "Scale Free Topology Model Fit,signed R^2",
    type = "n", main = paste("Scale independence"))
text(sft$fitIndices[, 1], -sign(sft$fitIndices[, 3]) * sft$fitIndices[,
    2], labels = powers, cex = cex1, col = "blue")
# this line corresponds to using an R^2 cut-off of h
abline(h = 0.9, col = "red")
# Mean connectivity as a function of the soft-thresholding
# power
plot(sft$fitIndices[, 1], sft$fitIndices[, 5], xlab = "Soft Threshold (power)",
    ylab = "Mean Connectivity", type = "n", main = paste("Mean connectivity"))
text(sft$fitIndices[, 1], sft$fitIndices[, 5], labels = powers,
    cex = cex1, col = "blue")
```

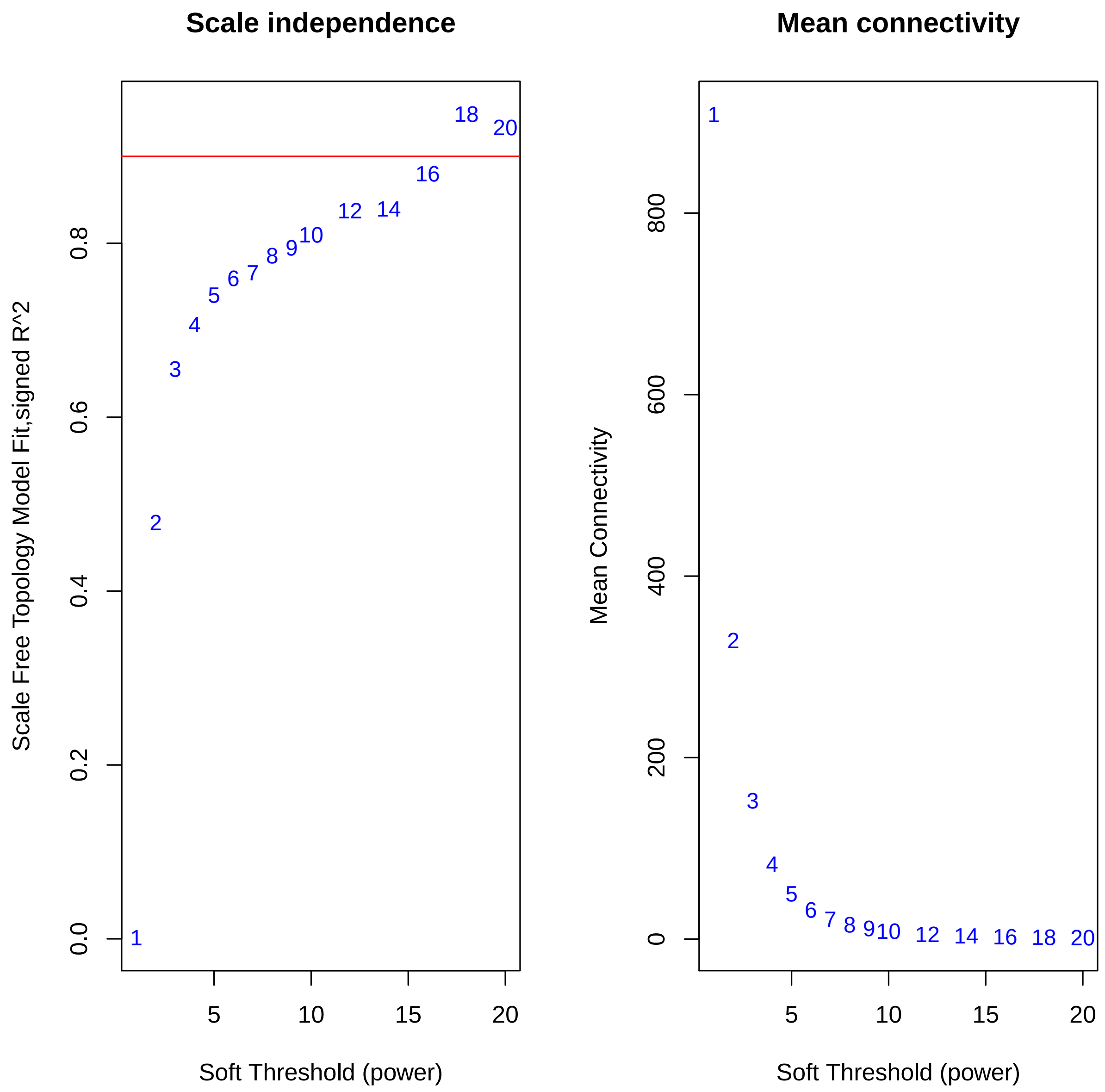

Creating modules based on threshold power

```
datExpr = Metdata
net = blockwiseModules(datExpr, power = 12, mergeCutHeight = 0.3,
    corType = "bicor", TOMType = "signed", minModuleSize = 5,
    reassignThreshold = 0, numericLabels = TRUE, saveTOMs = TRUE,
    minKMEtoStay = 0.5, minCoreKME = 0.5, minCoreKMESize = 3,
    saveTOMFileBase = "Metabolites_43 TOM", deepSplit = 2, verbose = 3)
```

```
##  Calculating module eigengenes block-wise from all genes
##    Flagging genes and samples with too many missing values...
##     ..step 1
```

```
##  ..Working on block 1 .
##     TOM calculation: adjacency..
##     ..will use 8 parallel threads.
##      Fraction of slow calculations: 0.000000
##     ..connectivity..
##     ..matrix multiplication (system BLAS)..
##     ..normalization..
##     ..done.
##    ..saving TOM for block 1 into file Metabolites_43 TOM-block.1.RData
##  ....clustering..
##  ....detecting modules..
##  ....calculating module eigengenes..
##  ....checking kME in modules..
##      ..removing 1 genes from module 1 because their KME is too low.
##      ..removing 1 genes from module 8 because their KME is too low.
##      ..removing 1 genes from module 12 because their KME is too low.
##      ..removing 1 genes from module 47 because their KME is too low.
##      ..removing 1 genes from module 82 because their KME is too low.
##      ..removing 1 genes from module 94 because their KME is too low.
##      ..removing 1 genes from module 105 because their KME is too low.
##      ..removing 1 genes from module 115 because their KME is too low.
##  ..merging modules that are too close..
##      mergeCloseModules: Merging modules whose distance is less than 0.3
##        Calculating new MEs...
```

The number of modules and metabolites within / 0 = unclustered modules

```r
table(net$colors)
```

```
##
##    0    1    2    3    4    5    6    7    8    9   10   11   12   13   14   15
## 1495  661  356  332  288  248   84   47   47   38   36   35   33   28   20   16
##   16   17   18   19   20   21   22   23   24   25   26   27   28   29   30   31
##   15   15   13   13   12   12   11   10   10    9    9    9    9    9    8    8
##   32   33   34   35   36   37   38   39   40   41   42   43   44   45   46   47
##    8    8    8    8    8    8    7    7    7    7    7    6    6    6    6    5
##   48   49   50   51
##    5    5    5    5
```

Plot Dendrogram

```r
# Convert labels to colors for plotting
mergedColors = labels2colors(net$colors)
# Plot the dendrogram with module colors below
plotDendroAndColors(net$dendrograms[[1]], mergedColors[net$blockGenes[[1]]],
    "Module colors", dendroLabels = FALSE, hang = 0.03, addGuide = TRUE,
    guideHang = 0.05)
```

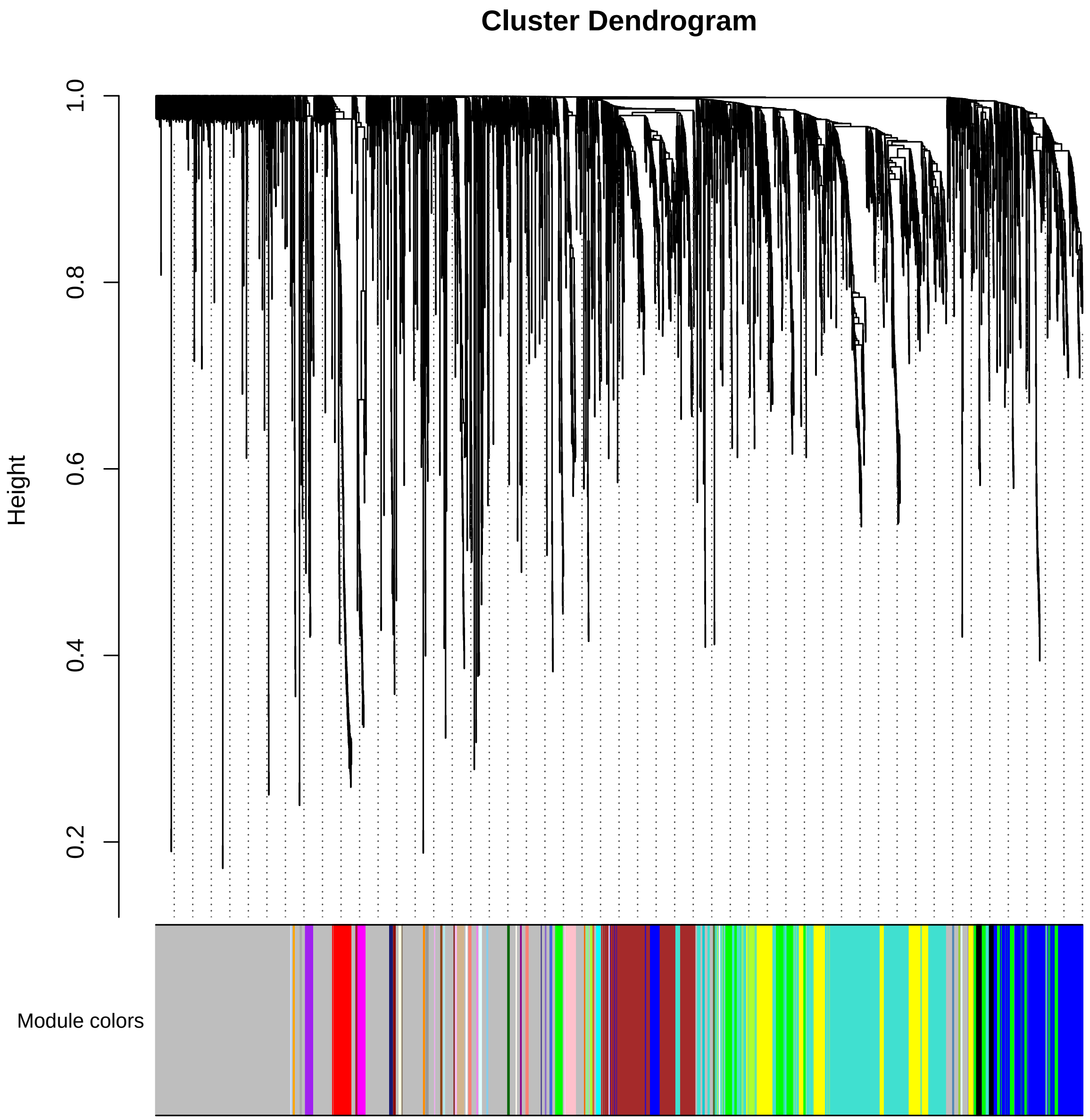

Module and module eigengene assignment

```
moduleLabels = net$colors
moduleColors = labels2colors(net$colors)
MEs = net$MEs
geneTree = net$dendrograms[[1]]
```

Relating modules to clinical traits

```r
# Relating modules to clinical traits Define numbers of genes
# and samples
nGenes = ncol(datExpr)
nSamples = nrow(datExpr)
# Recalculate MEs with color labels Eigengene placement along
# the PC1 axis of the modules correlated to clinical trait of
# choice
MEs0 = moduleEigengenes(datExpr, moduleColors)$eigengenes
MEs = orderMEs(MEs0)

# Correlating to trait (spearman)
moduleTraitCor = cor(MEs, dataTraits2, use = "p", method = c("spearman"))
moduleTraitPvalue = corPvalueStudent(moduleTraitCor, nSamples)

# FDR correcting Pvalues
moduleTraitPvalue_fdr <- as.data.frame(moduleTraitPvalue)
head(moduleTraitPvalue_fdr)
moduleTraitPvalue_fdr$Final_Weight <- p.adjust(moduleTraitPvalue_fdr$Final_Weight,
    method = "fdr")
moduleTraitPvalue_fdr$Fat_Glycogen <- p.adjust(moduleTraitPvalue_fdr$Fat_Glycogen,
    method = "fdr")
moduleTraitPvalue_fdr$Triglycerides <- p.adjust(moduleTraitPvalue_fdr$Triglycerides,
    method = "fdr")
moduleTraitPvalue_fdr <- as.matrix(moduleTraitPvalue_fdr)

# Plotting the spearman correlations of your modules with the
# clinical trait and apply FDR corrected p-value.  Will
# display correlations and their p-values
textMatrix = paste(signif(moduleTraitCor, 2), "\n(", signif(moduleTraitPvalue_fdr,
    1), ")", sep = "")
dim(textMatrix) = dim(moduleTraitCor)
par(mar = c(6, 8.5, 3, 3))
# Display the correlation values within a heatmap plot
labeledHeatmap(Matrix = moduleTraitCor, xLabels = names(dataTraits2),
    yLabels = names(MEs), ySymbols = names(MEs), colorLabels = FALSE,
    colors = blueWhiteRed(50), textMatrix = textMatrix, setStdMargins = FALSE,
    cex.text = 0.3, zlim = c(-1, 1), main = paste("Module-clinical trait relationships"))
```

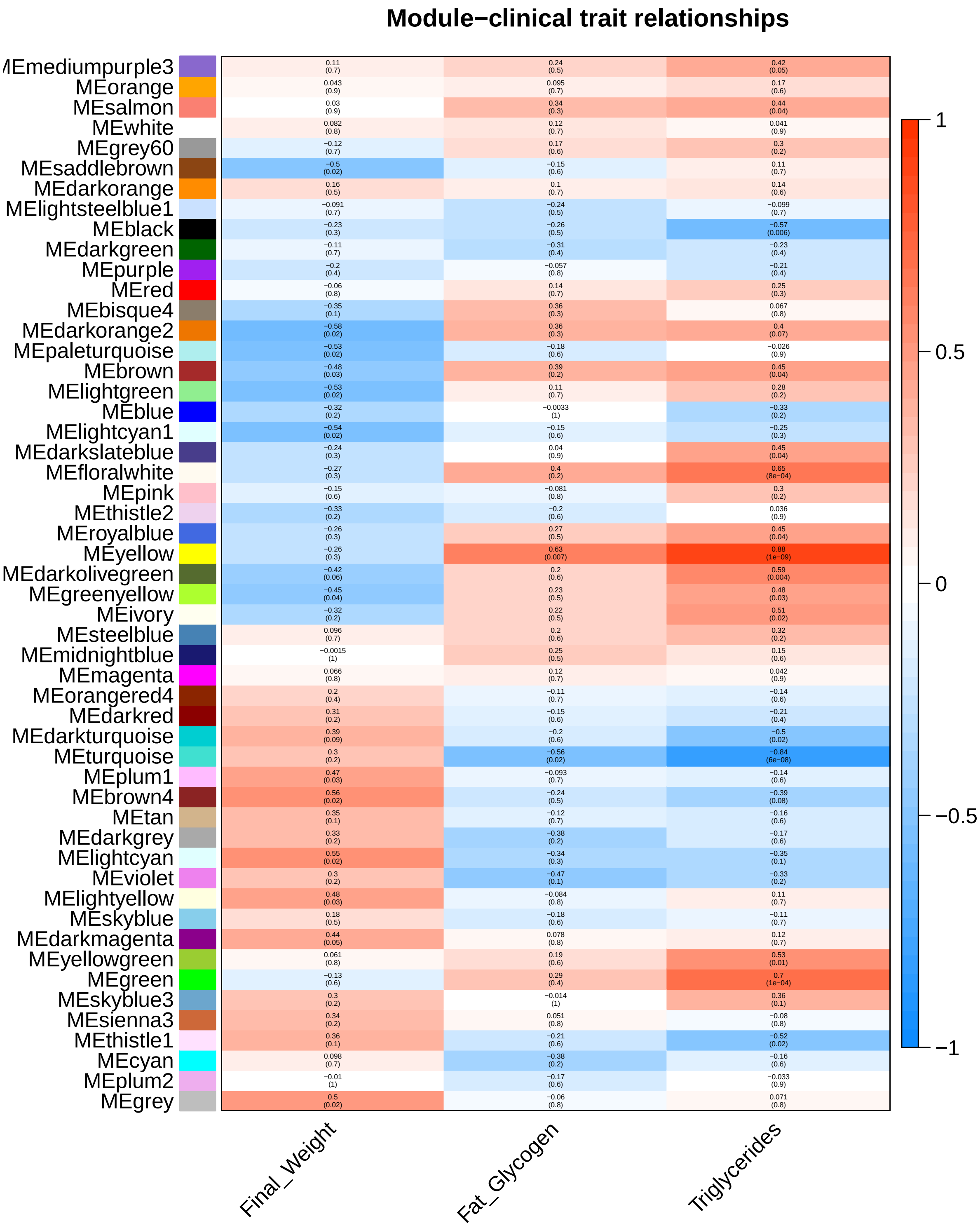
Module−clinical trait relationships
MEmediumpurple3
MEorange
MEsalmon
MEwhite
MEgrey60
MEsaddlebrown
MEdarkorange
MElightsteelblue1
MEblack
MEdarkgreen
MEpurple
MEred
MEbisque4
MEdarkorange2
MEpaleturquoise
MEbrown
MElightgreen
MEblue
MElightcyan1
MEdarkslateblue
MEfloralwhite
MEpink
MEthistle2
MEroyalblue
MEyellow
MEdarkolivegreen
MEgreenyellow
MEivory
MEsteelblue
MEmidnightblue
MEmagenta
MEorangered4
MEdarkred
MEdarkturquoise
MEturquoise
MEplum1
MEbrown4
MEtan
MEdarkgrey
MElightcyan
MEviolet
MElightyellow
MEskyblue
MEdarkmagenta
MEyellowgreen
MEgreen
MEskyblue3
MEsienna3
MEthistle1
MEcyan
MEplum2
MEgrey
Final_Weight
Fat_Glycogen
Triglycerides
1
0.5
0
−0.5
−1

Visualization 1: correlation plot with sizes, colors, and statistics

```
corrplot(moduleTraitCor, is.corr = FALSE, order = "original",
    method = "circle", tl.col = "black", cl.cex = 0.8, outline = TRUE,
    cl.ratio = 1, cl.align.text = "l", cl.offset = 0.5, tl.cex = 0.8,
    tl.pos = "lt", rect.lwd = 6, p.mat = moduleTraitPvalue_fdr,
    sig.level = 0.05, insig = "label_sig", col = colorRampPalette(c("navy",
        "white", "darkred"))(200))
```

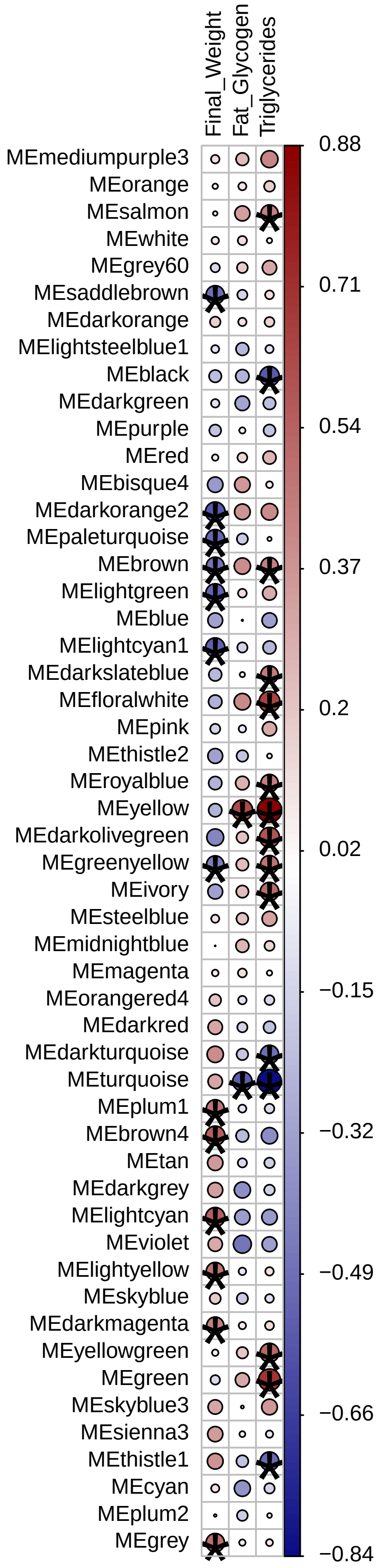
Final_Weight
Fat_Glycogen
Triglycerides
MEmediumpurple3
MEorange
MEsalmon
MEwhite
MEgrey60
MEsaddlebrown
MEdarkorange
MElightsteelblue1
MEblack
MEdarkgreen
MEpurple
MEred
MEbisque4
MEdarkorange2
MEpaleturquoise
MEbrown
MElightgreen
MEblue
MElightcyan1
MEdarkslateblue
MEfloralwhite
MEpink
MEthistle2
MEroyalblue
MEyellow
MEdarkolivegreen
MEgreenyellow
MEivory
MEsteelblue
MEmidnightblue
MEmagenta
MEorangered4
MEdarkred
MEdarkturquoise
MEturquoise
MEplum1
MEbrown4
MEtan
MEdarkgrey
MElightcyan
MEviolet
MElightyellow
MEskyblue
MEdarkmagenta
MEyellowgreen
MEgreen
MEskyblue3
MEsienna3
MEthistle1
MEcyan
MEplum2
MEgrey
0.88
0.71
0.54
0.37
0.2
0.02
−0.15
−0.32
−0.49
−0.66
−0.84

Relating modules to groups

```
# Correlating to group using binary table (spearman)
moduleTraitCorB = cor(MEs, groupTraits, use = "p", method = c("spearman"))
moduleTraitPvalueB = corPvalueStudent(moduleTraitCorB, nSamples)

# FDR correcting Pvalues
moduleTraitPvalue_fdrB <- as.data.frame(moduleTraitPvalueB)
head(moduleTraitPvalue_fdrB)
moduleTraitPvalue_fdrB$`C-MBGvCON` <- p.adjust(moduleTraitPvalue_fdrB$`C-MBGvCON`,
    method = "fdr")
moduleTraitPvalue_fdrB$MBGvCON <- p.adjust(moduleTraitPvalue_fdrB$MBGvCON,
    method = "fdr")
moduleTraitPvalue_fdrB$`MBG-RvCON` <- p.adjust(moduleTraitPvalue_fdrB$`MBG-RvCON`,
    method = "fdr")
moduleTraitPvalue_fdrB$`MBG-RvMBG` <- p.adjust(moduleTraitPvalue_fdrB$`MBG-RvMBG`,
    method = "fdr")
moduleTraitPvalue_fdrB$`MBGvC-MBG` <- p.adjust(moduleTraitPvalue_fdrB$`MBGvC-MBG`,
    method = "fdr")
moduleTraitPvalue_fdrB$Start_Mal_Diet <- p.adjust(moduleTraitPvalue_fdrB$Start_Mal_Diet,
    method = "fdr")
moduleTraitPvalue_fdrB$End_Mal_Diet <- p.adjust(moduleTraitPvalue_fdrB$End_Mal_Diet,
    method = "fdr")
moduleTraitPvalue_fdrB <- as.matrix(moduleTraitPvalue_fdrB)

# Plotting the spearman correlations of your modules with the
# clinical trait and apply FDR corrected p-value. Will
# display correlations and their p-values
textMatrix = paste(signif(moduleTraitCorB, 2), "\n(", signif(moduleTraitPvalue_fdrB,
    1), ")", sep = "")
dim(textMatrix) = dim(moduleTraitCorB)
par(mar = c(6, 8.5, 3, 3))
# Display the correlation values within a heatmap plot
labeledHeatmap(Matrix = moduleTraitCorB, xLabels = names(groupTraits),
    yLabels = names(MEs), ySymbols = names(MEs), colorLabels = FALSE,
    colors = blueWhiteRed(50), textMatrix = textMatrix, setStdMargins = FALSE,
    cex.text = 0.3, zlim = c(-1, 1), main = paste("Module-group relationships"))
```

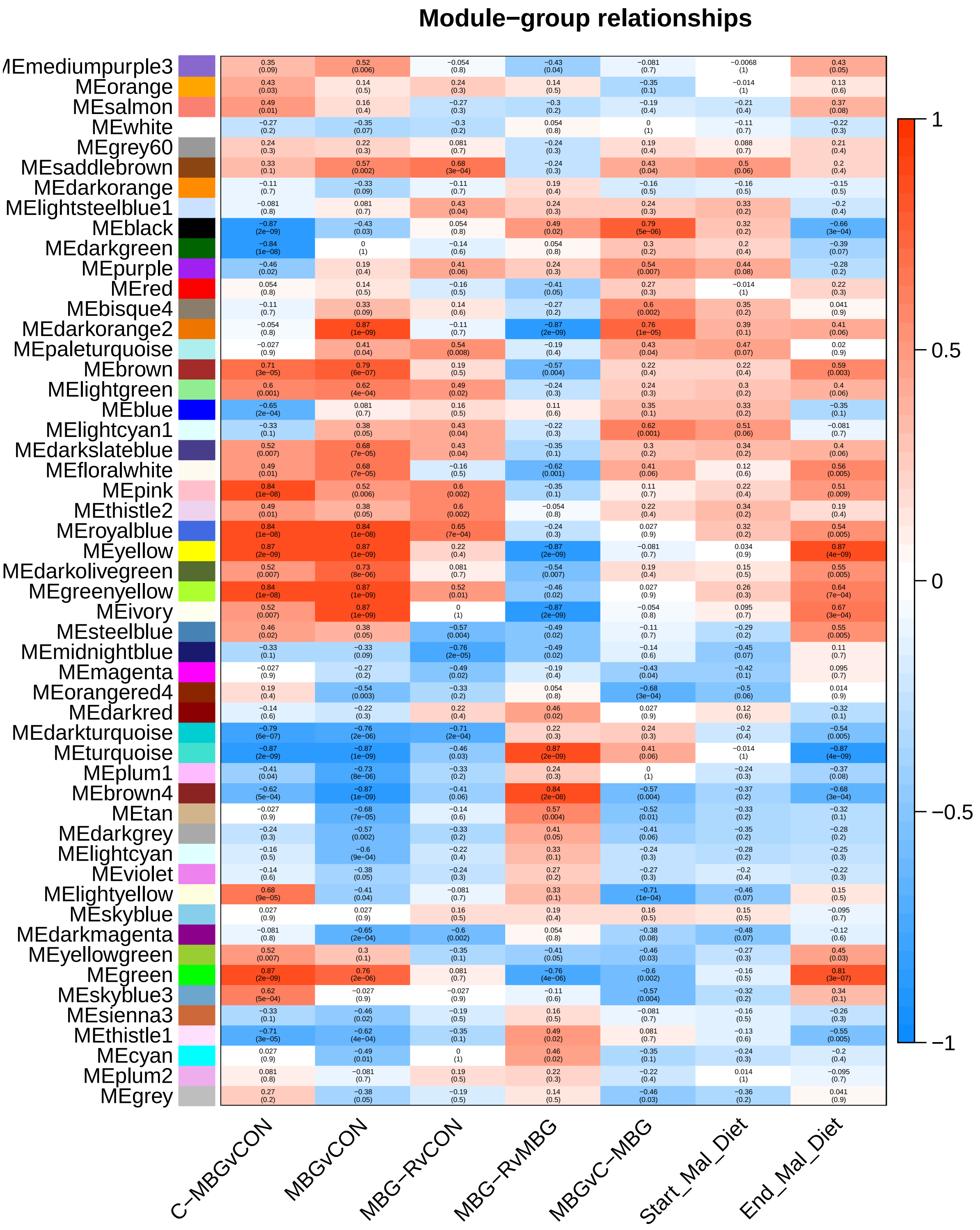
Module−group relationships
IEmediumpurple3
MEorange
MEsalmon
MEwhite
MEgrey60
MEsaddlebrown
MEdarkorange
MElightsteelblue1
MEblack
MEdarkgreen
MEpurple
MEred
MEbisque4
MEdarkorange2
MEpaleturquoise
MEbrown
MElightgreen
MEblue
MElightcyan1
MEdarkslateblue
MEfloralwhite
MEpink
MEthistle2
MEroyalblue
MEyellow
MEdarkolivegreen
MEgreenyellow
MEivory
MEsteelblue
MEmidnightblue
MEmagenta
MEorangered4
MEdarkred
MEdarkturquoise
MEturquoise
MEplum1
MEbrown4
MEtan
MEdarkgrey
MElightcyan
MEviolet
MElightyellow
MEskyblue
MEdarkmagenta
MEyellowgreen
MEgreen
MEskyblue3
MEsienna3
MEthistle1
MEcyan
MEplum2
MEgrey
C−MBGvCON
MBGvCON
MBG−RvCON
MBG−RvMBG
MBGvC−MBG
Start_Mal_Diet
End_Mal_Diet
1
0.5
0
−0.5
−1

Visualization 2: correlation plot with sizes, colors, and statistics

```
corrplot(moduleTraitCorB, is.corr = FALSE, order = "original",
    method = "circle", tl.col = "black", cl.cex = 0.8, outline = TRUE,
    cl.ratio = 1, cl.align.text = "l", cl.offset = 0.5, tl.cex = 0.8,
    tl.pos = "lt", rect.lwd = 6, p.mat = moduleTraitPvalue_fdrB,
    sig.level = 0.05, insig = "label_sig", col = colorRampPalette(c("navy",
        "white", "darkred"))(200))
```

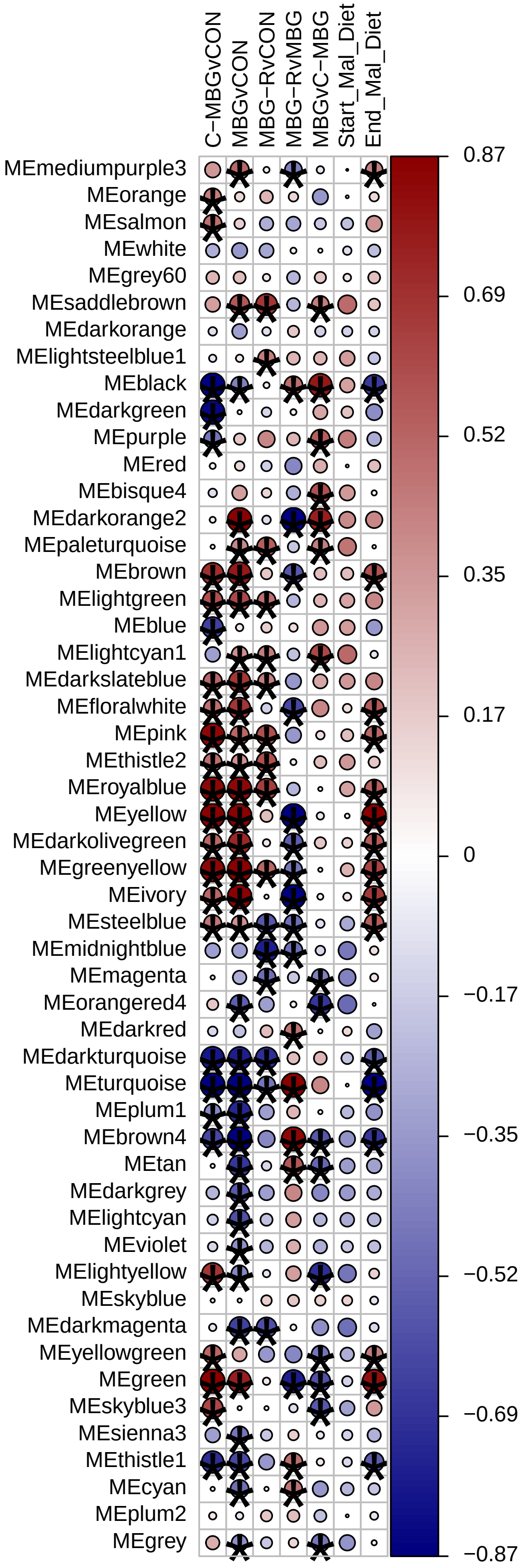

C−MBGvCON
MBGvCON
MBG−RvCON
MBG−RvMBG
MBGvC−MBG
Start_Mal_Diet
End_Mal_Diet
MEmediumpurple3
MEorange
MEsalmon
MEwhite
MEgrey60
MEsaddlebrown
MEdarkorange
MElightsteelblue1
MEblack
MEdarkgreen
MEpurple
MEred
MEbisque4
MEdarkorange2
MEpaleturquoise
MEbrown
MElightgreen
MEblue
MElightcyan1
MEdarkslateblue
MEfloralwhite
MEpink
MEthistle2
MEroyalblue
MEyellow
MEdarkolivegreen
MEgreenyellow
MEivory
MEsteelblue
MEmidnightblue
MEmagenta
MEorangered4
MEdarkred
MEdarkturquoise
MEturquoise
MEplum1
MEbrown4
MEtan
MEdarkgrey
MElightcyan
MEviolet
MElightyellow
MEskyblue
MEdarkmagenta
MEyellowgreen
MEgreen
MEskyblue3
MEsienna3
MEthistle1
MEcyan
MEplum2
MEgrey
0.87
0.69
0.52
0.35
0.17
0
−0.17
−0.35
−0.52
−0.69
−0.87

To obtain a list of metabolites within each module...also correlates module members with trait of choice / Useful to analyse red, yellow, and turquoise modules

```
Fat_Glycogen = as.data.frame(dataTraits2$Fat_Glycogen)
names(Fat_Glycogen) = "Fat_Glycogen"
# names (colors) of the modules
modNames = substring(names(MEs), 3)
# definine module membership of each metabolite and its
# significance
geneModuleMembership = as.data.frame(cor(datExpr, MEs, use = "p"))
MMPvalue = as.data.frame(corPvalueStudent(as.matrix(geneModuleMembership),
    nSamples))
names(geneModuleMembership) = paste("MM", modNames, sep = "")
names(MMPvalue) = paste("p.MM", modNames, sep = "")

# correlating each metabolite to the trait
geneTraitSignificance = as.data.frame(cor(datExpr, Fat_Glycogen,
    use = "p", method = c("spearman")))
GSPvalue = as.data.frame(corPvalueStudent(as.matrix(geneTraitSignificance),
    nSamples))
names(geneTraitSignificance) = paste("GS.", names(Fat_Glycogen),
    sep = "")
names(GSPvalue) = paste("p.GS.", names(Fat_Glycogen), sep = "")

# combining correlation, significance, and membership with
# metabolites and modules
Metabolite = names(datExpr)

MetInfo = data.frame(Metabolite = Metabolite, moduleColor = moduleColors,
    geneTraitSignificance, GSPvalue)
# order modules by significance for Atopy
modOrder = order(-abs(cor(MEs, Fat_Glycogen, use = "p")))

# Add module membership information in the chosen order
for (mod in 1:ncol(geneModuleMembership)) {
    oldNames = names(MetInfo)
    MetInfo = data.frame(MetInfo, geneModuleMembership[, modOrder[mod]],
        MMPvalue[, modOrder[mod]])
    names(MetInfo) = c(oldNames, paste("MM.", modNames[modOrder[mod]],
        sep = ""), paste("p.MM.", modNames[modOrder[mod]], sep = ""))
}

# Order the genes in the geneInfo variable first by module
# color, then by geneTraitSignificance
MetOrder = order(MetInfo$moduleColor, -abs(MetInfo$GS.Fat_Glycogen))
MetInfo = MetInfo[MetOrder, ]

# This table shows who is clustering in each module and how
# they relate to the trait.
write.csv(MetInfo, file = "MetInfo_FG_spearman.csv")

# This table contains the MEs and the colors
write.csv(MEs, file = "Modular Eigengene")
```
